# The Microbial Ecology of Antarctic Sponges

**DOI:** 10.1007/s00248-025-02543-y

**Published:** 2025-05-17

**Authors:** Qi Yang, Rachel Downey, Jonathan S. Stark, Glenn J. Johnstone, James G. Mitchell

**Affiliations:** 1 CSIRO Agriculture and Food, Urrbrae, SA 5064 Australia; 2 College of Science and Engineering, Flinders University, Bedford Park, SA 5042 Australia; 3 Fenner School of Environment & Society, Australian National University, Canberra, ACT 2601 Australia; 4 East Antarctic Monitoring Program, Australian Antarctic Division, Kingston, TAS 7050 Australia

**Keywords:** Antarctic sponges, Microbiome, Symbiosis, Holobiont resilience, Polar benthic ecosystems

## Abstract

Microbial communities in Antarctic marine sponges have distinct taxonomic and functional profiles due to low temperatures, seasonal days and nights, and geographic isolation. These sponge holobionts contribute to nutrient cycling, structural habitat formation, and benthic ecosystem resilience. We review Antarctic sponge holobiont knowledge, integrating culture-based and molecular data across environmental and taxonomic gradients. Although microbiome data exist for only a fraction of the region’s 593 known sponge species, these hosts support diverse symbionts spanning at least 63 bacterial, 5 archaeal, and 6 fungal phyla, highlighting the complexity and ecological significance of these understudied polar microbiomes. A conserved core microbiome, dominated by Proteobacteria, Bacteroidetes, Nitrospinae, and Planctomycetes, occurs across Antarctic sponges, alongside taxa shaped by host identity, depth, and environment. Metagenomic data indicate microbial nitrogen cycling, chemoautotrophic carbon fixation, and stress tolerance. Despite these advances, major knowledge gaps remain, particularly in deep-sea and sub-Antarctic regions, along with challenges in taxonomy, methodological biases, and limited functional insights. We identify key research priorities, including developing standardised methodologies, expanded sampling across ecological and depth gradients, and integrating multi-omics with environmental and host metadata. Antarctic sponge holobionts provide a tractable model for investigating microbial symbiosis, functional adaptation, and ecosystem processes in one of Earth’s most rapidly changing marine environments.

**Supplementary Information:**

The online version contains supplementary material available at 10.1007/s00248-025-02543-y.

## Introduction

Microbial symbioses are central to the structure and function of marine ecosystems, playing key roles in nutrient cycling, stress tolerance, and host fitness. Around Antarctica, microbes and the ancient marine sponges form stable holobionts [1]. These have evolved under low temperatures, seasonal days and nights, and prolonged geographic isolation, resulting in distinct patterns of microbial diversity, specificity, and functional adaptation [2]. Antarctic sponge holobionts are key components of benthic ecosystems. They provide habitat complexity and are major trophic networks. Recognised as indicators of vulnerable marine ecosystems by the Commission for the Conservation of Antarctic Marine Living Resources (CCAMLR) [3], sponge holobionts exist from coastal shelves and subtidal zones to deep-sea basins and hydrothermal vents [4–9].

All sponge classes are present, with Demospongiae being particularly species-rich [4, 5, 10–14]. Microbiome studies exist for less than 10% of the species. These studies reveal at least 63 bacterial phyla, along with archaeal and fungal phyla [15–17]. Antarctic sponge microbiomes differ markedly from tropical and temperate microbiomes by displaying low microbial abundance, reduced taxonomic complexity, and high host specificity [18–21]. Dominant groups such as Proteobacteria, Bacteroidetes, Nitrospinae, and Planctomycetes form a conserved core microbiome, with variation in peripheral taxa driven by host identity, depth, and local environmental conditions [19, 22–27]. Recent metagenomic studies have identified functional traits linked to survival in polar ecosystems, including genes for nitrogen cycling, chemoautotrophic carbon fixation, and oxidative stress response [8, 18, 19]. These functions highlight the role of microbial symbionts in mediating host adaptation to nutrient-poor, light-limited, and cold environments. Furthermore, core microbial taxa demonstrate notable stability across environmental gradients, suggesting tight host-microbe co-evolution and filtering mechanisms.

These distinct microbial features underscore the need for targeted investigations into polar sponge holobionts, where knowledge remains limited compared to temperate and tropical systems. A comprehensive literature search was conducted using Web of Science, Scopus, and Google Scholar with keywords such as Antarctic, deep sea, sponge, porifera, symbionts, holobiont, microbiome, and other related terms. The selection criteria were: (1) peer-reviewed research published in the last 20 years; (2) studies that applied culture-dependent or molecular methods (e.g., amplicon sequencing, metagenomics) to assess microbial communities were included; and (3) studies focusing solely on single-strain genome sequencing or natural product isolation without microbial community analysis were excluded. Sponge records from Ocean Biodiversity Information System (OBIS), The Register of Antarctic Marine Species (RAMS), and four genetic databases were analysed for taxonomic accuracy, spatial coverage, and sampling bias within the CCAMLR boundary, with metadata curated and standardised via WoRMS to ensure the correct associations of microbial communities with sponge species.

Our key objectives were to (1) evaluate current research coverage and regional biases of holobionts, (2) enhance understanding of evolutionary and ecological factors shaping sponge-microbiome diversity, (3) assess potential threats to these symbiotic partnerships under changing environmental conditions, and (4) identify critical knowledge gaps and methodological challenges.

## Antarctic Microbiome Host Diversity

### Morphological Estimates

The 2023 RAMS list includes 513 species, subspecies, and varieties. This includes 132 new Demospongiae species, a doubling of Hexactinellida species from 29 to 65, and a fivefold rise in Calcarea from 12 to 69. Four species now represent Homoscleromorpha.

Patterns of sponge taxonomic richness across Antarctic genera directly affect the distribution and diversification of associated microbial communities within sponge holobionts. Genus-level analysis reveals that 69 sponge genera, nearly half of all reported, are monospecific, accounting for 13% of species richness. Fourteen percent of genera are half of described species, suggesting that microbial diversity may be disproportionately concentrated in a limited number of host lineages. These species-rich genera are affiliated with six Demospongiae families, with additional representation from Hexactinellida and Calcarea, highlighting key host clades for conserved and functionally distinct microbiomes.

About 67% of Antarctic sponge species are endemic (RAMS, WoRMS). This suggests prolonged evolutionary host isolation, suggesting concomitant specialised microbial symbionts. Complementary OBIS data document 537 sponge species from over 31,000 CCAMLR (Supplementary Table 1) records. However, OBIS and RAMS discrepancies indicate cryptic species diversity. These host-level uncertainties have downstream consequences for microbial ecology, as unresolved taxonomy and inconsistent metadata limit accurate assessments of microbiome specificity, distribution, and functional integration across Antarctic sponge holobionts. Conversely, 12% of RAMS-verified species are absent from OBIS.

Despite the growth in species-level records, the proportion of fully identified specimens has declined, from ~ 63% to under 50%, highlighting a drop in taxonomic resolution over time [4]. This trend is most evident in Hexactinellida (< 60% identified), with higher resolution retained in Calcarea and Demospongiae (> 70%). These findings indicate the continued need for taxonomic standardisation and improved identification protocols in Antarctic sponge biodiversity surveys.

### Genetic Estimates

Molecular data for Antarctic sponge diversity is sparse. Across the BOLD, GenBank, EMBL-EBI, and SBD databases, only 80 morphospecies are represented by sequence data (Supplementary Table 2), covering ~ 17% of verified species [28–31]. Demospongiae account for ~ 80% of records, with Hexactinellida (~ 19%) and Calcarea (~ 4%) underrepresented. Most identifications rely on mitochondrial COI markers (Supplementary Table 3), while comprehensive multilocus data are primarily restricted to Hexactinellida [12, 32]. This limited molecular framework hampers efforts to resolve host-specific microbial associations and interpret evolutionary patterns in sponge holobionts (Supplementary Fig. 1).

These datasets reveal in situ evolution in several lineages [33–35], limited but notable gene flow across large spatial scales [36], and regionally unique phylogenetic trajectories [37, 38]. However, unresolved taxonomy and morphological–genetic incongruence remains common [34, 35], and no cryptic species have been confirmed, in contrast to many other Antarctic taxa [39–41]. Such taxonomic uncertainty complicates the delineation of microbial specificity and the detection of co-diversification signals within sponge-microbe systems.

## Microbial Diversity in Antarctic Sponges

Our understanding of these communities has benefitted from modern high-throughput sequencing techniques, which reveal the full breadth of microbial diversity. These approaches have uncovered remarkable diversity across bacterial, archaeal, and fungal communities. Reviewed studies document 63 bacterial phyla, 5 archaeal phyla, and 6 fungal phyla. Culture-dependent studies have focused on 28 Antarctic species from 23 genera, 17 families, and 6 orders in 2 classes (Supplementary Table 4), while molecular approaches have expanded coverage to 35 species from 25 genera, 19 families, and 9 orders in 3 Porifera classes (Supplementary Table 5). This combined approach has revealed that Antarctic sponges likely belong to the low microbial abundance group, typically hosting between 10^5^ and 10^6^ bacteria per gram of sponge tissue, comparable to microbial abundances found in seawater [23, 24, 42]. These microbiomes exhibit a specific signature, less complex (in terms of alpha-diversity) and more heterogeneous (in terms of beta-diversity) [26], when compared to the microbiome of temperate and tropical sponges [43]. The sponge status is found to be the primary factor driving microbial variability in deep-sea sponges, followed by location, host phylogeny, and environmental cluster. We believe the ecological context of host-microbe associations would be the key in order to comprehensively understand the patterns and drivers of microbial composition and structure.

### Culture-Dependent Insights into Microbial Diversity

**Fig. 1:**
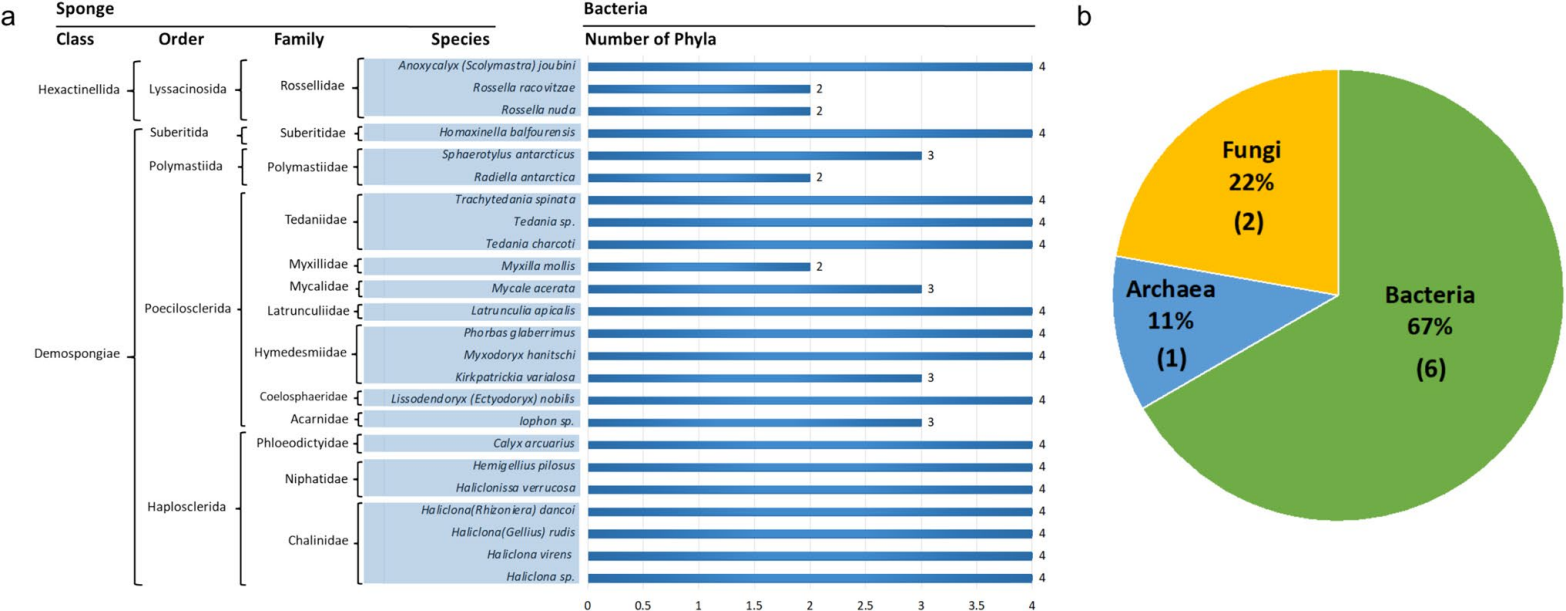
Diversity and composition of microbial communities associated with Antarctic sponges based on culture-dependent analyses. **a** Number of bacterial phyla identified from each sponge species, highlighting variability in microbial associations (see Supplementary Table 4). **b** Distribution of identified microbial phyla across the three domains of life (Bacteria, Archaea, Eukarya); the number of phyla within each domain is shown in parentheses

Culture-based approaches have provided fundamental insights into the cultivatable portion of Antarctic sponge microbiomes. The Bacteroidetes, Nitrospinae, Planctomycetes, and Proteobacteria occur in all Antarctic sponges. The Acidobacteria, Actinobacteria, Firmicutes, Spirochaetes, and Verrucomicrobia are found at nine locations. There are distinct patterns of bacterial, archaeal, and fungal diversity (Supplementary Table 4). Prokaryotic diversity has been measured in 24 species (Fig. 1a) and eukaryotic diversity in 5 species [15, 44, 45].

Bacterial isolates come from Actinobacteria, Bacteroidetes, Bacillota, Firmicutes, Planctomycetes and Proteobacteria (Fig. 1b). Gamma and Alphaproteobacteria and the *Cytophaga*/*Flavobacterium* group of Bacteroidetes, with additional representation from Firmicutes and Planctomycetes, were dominant in five Ross Sea Demospongiae species (*Kirkpatrick variolosa*, *Latrunculia apicalis*, *Homaxinella balfourensis*, *Mycale acerate*, and *Sphaerotylus antarcticus*) [46]. More recent Ross Sea investigations [15] revealed another 11 sponge species and indicated the shifts in these patterns, with Gammaproteobacteria, Actinobacteria, and Bacteroidetes dominating, while Alphaproteobacteria and Firmicutes showed reduced representation.

Cultivation studies have yielded significant insights into functional diversity. Antimicrobial compound screening identified 140 bacterial strains from 15 genera, with three Antarctic Demospongiae species showing potential for novel antimicrobial compounds, particularly volatile organic compounds [27]. Studies of psychotropic bacteria have shown increasing success in isolation efforts, from 37 to 105 strains, primarily representing Proteobacteria, the CFB (*Cytophaga*, *Fusobacterium*, and *Bacteroides*) group, and Actinobacteria [47–49]. Gammaproteobacteria consistently dominated these isolates, with fewer representatives from Alphaproteobacteria and the CFB group, including the unique sequence from the ecologically relevant genus *Polaribacter*. Notably, *Polaribacter* remains an active genus, with taxonomic revisions and new species continuing to be described as recently as 2024, underscoring the dynamic nature of microbial classification in polar environments. Deep-sea sponges have yielded particularly interesting findings, with 46 g-positive strains identified from Firmicutes and Actinobacteria, including the first isolation of strains from *Dietzia* and *Brevibacterium* [22]. Given the recent redefinition of species within *Brevibacterium,* such findings highlight the potential for discovering undescribed taxa and refining our understanding of sponge-associated microbial diversity. These psychrotolerant and taxonomically diverse bacteria also offer promising avenues for bioprospecting, particularly in the search for cold-adapted enzymes, novel metabolites, and antimicrobial compounds suited to extreme environments [50, 51]. Genome sequencing of selected bacterial isolates has further revealed the functional potential of cultivable symbionts. Three *Arthrobacter* strains isolated from different sponge species possess stress response and secondary metabolite genes [52], while novel members of *Sporosarcina* and *Nesterenkonia* from a single host show genomic features consistent with osmoregulation and environmental adaptation [8]. These findings highlight the value of cultivation and genome-based approaches for uncovering microbial contributions to sponge ecology.

Beyond bacteria, cultivation has recovered Crenarchaeota archaea and Ascomycota and Basidiomycota fungi. The limited fungal studies have identified cold-adapted symbionts with important insights. DGGE has shown diverse eukaryotic communities, including diatoms and dinoflagellates as part of holobionts [46]. The diatom cluster with closest phylogenetic relatives including *Chaetoceros rostratus* (93% homology), the Antarctic centric diatom *Thalassiosira antarctica* (97% homology), and *Paraphysomonas butcheri* (98% homology). The closest known dinoflagellates were *Lepidodinium viridae* (97% homology) and *Gyrodinium galatheanum* (95% homology). Subsequent culturing efforts yielded 20 psychrotolerant yeast isolates [44], over 100 fungal strains primarily assigned to Ascomycota [45], and additional yeasts from Basidiomycota [53]. Notably, 36 isolates could not be identified at the genus level, suggesting an undescribed diversity within polar sponge holobionts.

### Culture-Independent Insights into Microbiome Diversity

**Fig. 2:**
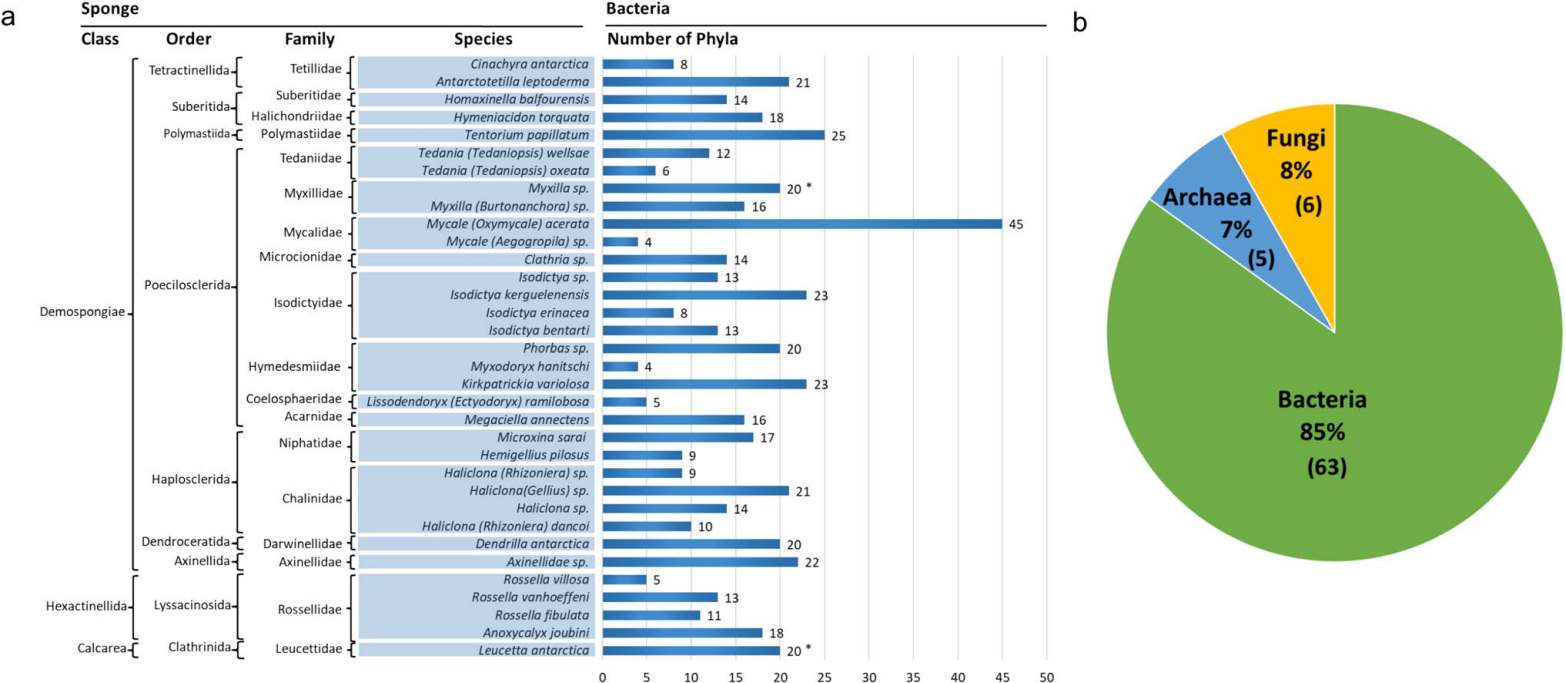
Diversity of microbial communities associated with Antarctic sponges using culture-independent analysis. **a** Number of bacterial phyla identified in each sponge species. The data were merged if the sponge species was analysed by multiple studies. **b** Distribution of microbial phyla across the three domains. Note: *Data from shotgun metagenomic sequencing include. *Sphaerotylus antarcticus* and *Cladorhiza* sp. are excluded due to unclear bacterial phyla counts in studies of Papale et al. [16] and Ruocco et al. [54], respectively. *Myxilla* (*Burtonanchora*) sp. and *Myxilla* sp. are presented separately, as they were analysed using 16S rRNA gene-based metagenomic sequencing and shotgun metagenomic sequencing, respectively (see Supplementary Table 5)

High-throughput sequencing, predominantly 16S rRNA gene-based amplicon sequencing, has dramatically expanded our understanding of Antarctic sponge microbiome diversity (Fig. 2 and Supplementary Table 5). These investigations spanned 35 sponge species. Sequencing has uncovered 63 bacterial phyla, 5 archaeal phyla, and 6 fungal phyla (Fig. 2b). The average number of microbial phyla per sponge species now exceeds 15 (Fig. 2a). The highest microbial diversity is in *Mycale* (*Oxymycale*) *acerata*, with 45 bacterial phyla [16, 18, 23, 42, 54].

Antarctic sponge holobiont complexity was first revealed with the inclusion of Bacteria, Archaea, and Eukaryota [43], identifying 25 bacterial and 2 archaeal phyla and 8 eukaryotic supergroups. The dominant taxa were Proteobacteria, Bacteroidetes, Verrucomicrobia, Thaumarchaeota, Planctomycetes, Euryarchaeota, Thaumarchaeota, Stramenopiles, Alveolata, and Hacrobia. Taxonomic analysis has revealed notable patterns of host specificity and microbial diversity. Poecilosclerida is the most extensively studied order [8, 18, 19, 26, 42, 55, 56]. *Mycale* (*Oxymycale*) *acerata* stands out, with 31 bacterial phyla, indicating significant microbial diversity [19]. Order Haplosclerida microbial diversity ranges from 6 to 21 [16, 23, 24, 43, 54, 57]. It is worth noting that diversity may vary with the reported taxonomic level. The orders Polymastiida, Suberitida, and Tetractinellida species host up to 25 bacterial phyla, again underscoring microbial community diversity (Supplementary Table 5). Conversely, singles studies of Axinellida and Dendroceratida still found 20 to 27 bacterial phyla, suggesting single studies can reveal the extent of microbial diversity [23, 25, 26]. Temporal analyses provided insights into microbial dynamics. *Mycale* (*Oxymycale*) *acerata* continued to be dominated by *Gammaproteobacteria* over the sampling time from 2016 to 2018, suggesting a tightly regulated symbiotic relationship between sponge hosts and their microbiomes [19].

### Culture-Independent Insights into Holobiont Ecology

Environmental factors such as depth and hydrothermal vent proximity are critical drivers of some core and secondary microbial community members. Deep-sea sponges hosted less complex but more heterogeneous microbiomes compared to their shallow-water counterparts [26], with depth changes influencing community structure [24]. Carnivorous sponges near hydrothermal vents harbored chemosynthetic *Gammaproteobacteria*, reflecting adaptations to nutrient-limited conditions [55]. Comparative studies investigating sponges from Antarctica and South America emphasised the role of environmental factors, such as variations in light, temperature, and nutrient availability, in driving microbiome diversity [58]. A recent global comparison highlighted strong habitat specificity in Antarctic sponge microbiomes, which exhibited lower alpha diversity, higher compositional similarity, and enrichment of habitat-specific taxa [59]. Keystone candidates such as *Nitrosomonas oligotropha*, *Candidatus Nitrosopumilus*, and *Polaribacter* were highly connected in co-occurrence networks, suggesting functional centrality. These patterns reflect the influence of environmental filtering and geographic isolation in shaping distinct, ecologically cohesive microbiomes in Antarctic sponges. Additionally, experiments exposing Antarctic sponges to controlled heat stress, which used aquaria set at local seawater temperature (control), moderate (4–5 °C above the control), and extreme (6–10 °C above the control) warming conditions based on climate change projections, revealed species-specific microbiome shifts while maintaining core microbial populations, indicating some resilience to warming conditions [60].

A high-resolution study of four Antarctic sponge species, documenting 27 bacterial phyla, revealed host-specific yet low-diversity microbial communities [43]. Despite geographic separation, each species maintained a stable core microbiome, with a small number of dominant OTUs (operational taxonomic unit) and clear divergence from surrounding seawater communities. OTU-level analysis insights suggest that key symbionts affiliated with Thaumarchaeota and Nitrospirae may contribute to nitrification, indicating a role in nitrogen cycling under extreme Antarctic conditions. These microbiomes appear to be structured by host identity, enriched with generalist taxa adapted to cold, oligotrophic environments.

To further explore microbial functions and resilience in polar ecosystems, shotgun metagenomic investigations revealed that the holobionts possess distinct and specialised metabolic capabilities, enriched in genes linked to symbiosis and key biogeochemical processes [8]. These include pathways for nitrogen cycling, such as ammonia and nitrite oxidation and denitrification, as well as chemoautotrophic, light-independent carbon fixation, supporting holobiont survival during prolonged periods of darkness. Functional features such as CRISPR systems, eukaryotic-like proteins, and mobile genetic elements further suggest adaptations to a stable symbiotic lifestyle. Dominated by Proteobacteria and Thaumarchaeota, with fungal contributions among eukaryotes, these communities also exhibit functional potential for sulfur and phosphorus cycling. Complementary findings from Moreno-Pino et al. [61] confirm such metabolic specialisation, underscoring the dominance of cold-adapted microbial lineages and their roles in elemental cycling and symbiotic stability across polar habitats.

These findings collectively underscore the critical ecological roles Antarctic sponge-associated microbes play in nutrient cycling and stress adaptation. Moreover, they highlight the untapped potential for biotechnological applications, including the discovery of novel bioactive compounds. By integrating taxonomic, functional, and temporal analyses, this review advances the understanding of microbial communities in Antarctic sponges and sets the stage for future research into their ecological and industrial significance.

### Core and Variable Components of Antarctic Sponge Microbiomes

**Fig. 3:**
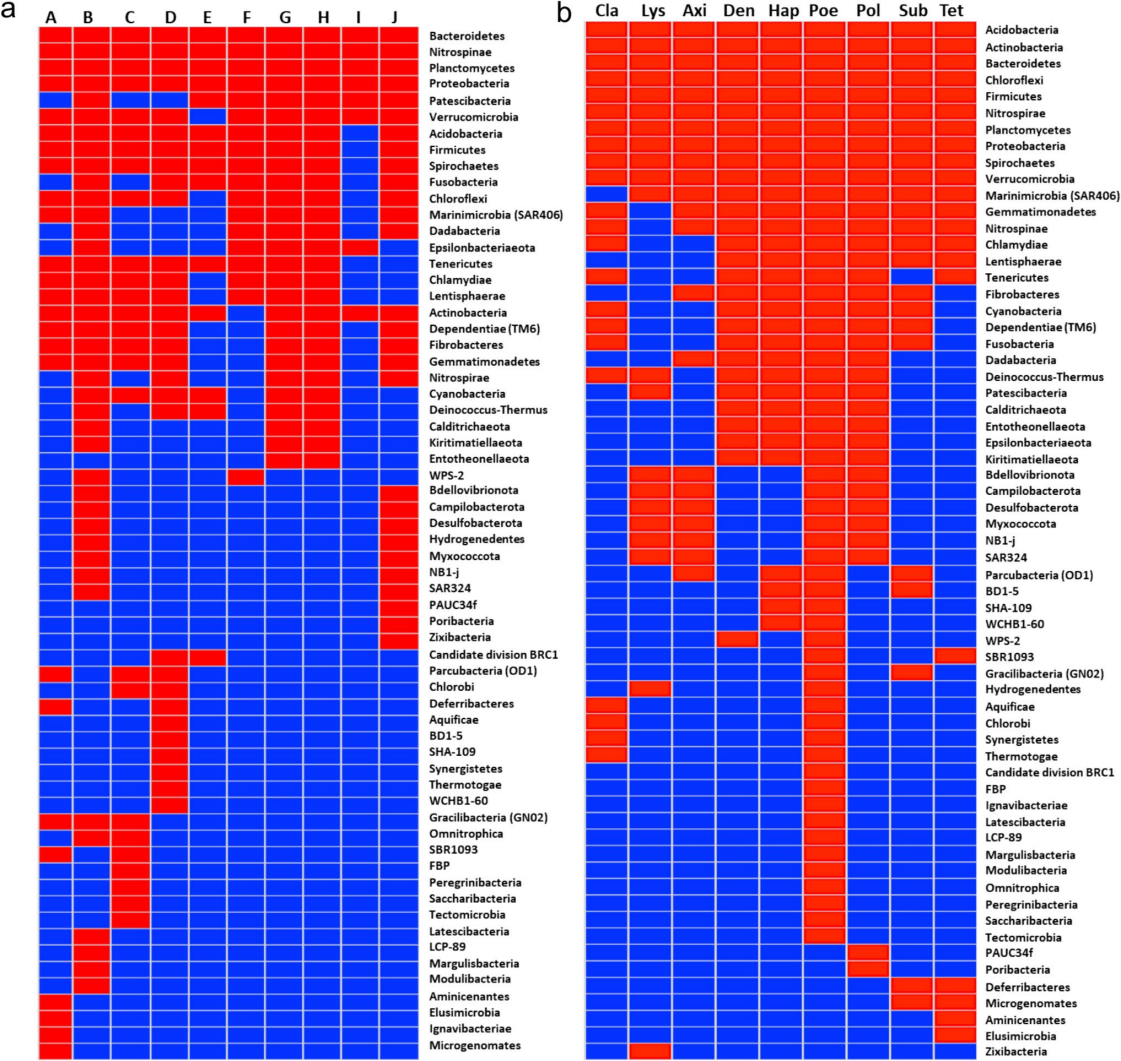
Distribution of bacterial taxa across various sponge orders and geographic locations. **a** The x-axis marks the locations. A: Antarctic shelf around the South Shetland Islands; B: Cape Kemp in Doumer Island, Palmer Archipelago, Western Antarctic Peninsula; C: Fildes Bay, King George Island and South Bay, Doumer Island, Palmer Archipelago, Antarctica; D: Fildes Bay, King George Island, South Shetlands, Antarctica; E: Terra Nova Bay (Ross Sea, Antarctica); F: Tethys Bay (Victoria Land, Antarctica); G: Whalers Bay, Deception Island; H: Whalers Bay, Deception Island, and Rothera Research Station, and Bernardo O’Higgins Research Station, Half Moon Island; I: East Scotia Ridge and Juan de Fuca Ridge, Southern Ocean; J: Weddell Sea Filchner-Ronne and Prince Gustav. **b** The sponge orders refer to, from left to right, Clathrinida Hartmann, 1958; Lyssacinosida Zittell, 1877; Axinellida; Dendroceratida Minchin, 1900; Haplosclerida Topsent, 1928; Poecilosclerida; Polymastiida Morrow & Cárdenas, 2015; Suberitida Chombard & Boury-Esnault, 1999; and Tetractinellida Marshall, 1876. The red blocks represent the presence of the microbial phyla, and the blue blocks represent the absence of the microbial phyla

Integrating culture-dependent and independent studies reveals clear patterns in Antarctic sponge microbiome composition, with distinct core and variable components across taxonomic scales. Four bacterial phyla emerged as universal core members shared across all studied Antarctic sponges: Bacteroidetes, Nitrospinae, Planctomycetes, and Proteobacteria (Fig. 3a). This core community is augmented in many sponges by Acidobacteria, Actinobacteria, Firmicutes, Spirochaetes, and Verrucomicrobia.

Host taxonomy strongly influenced microbiome structure, with clear patterns emerging at the order level. The Acidobacteria, Actinobacteria, Bacteroidetes, Chloroflexi, Firmicutes, Nitrospirae, Planctomycetes, Proteobacteria, Spirochaetes, and Verrucomicrobia were present in at least some species of all 10 sponge orders (Fig. 3b). The Marinimicrobia (SAR406 clade) and Gemmatimonadetes were prevalent but less universal. This taxonomic consistency suggested potential co-evolutionary relationships between hosts and their core microbiota.

The consistency of core taxa across host taxonomy and geographic locations pointed to fundamental roles for these microbes in Antarctic sponge biology. The significant overlap between predominant taxa across locations and orders (23 of 24 taxa shared) suggested strong selective pressures maintaining these core associations despite environmental variation. This stability in core microbiome components and host-specific taxa indicated complex and long-established symbiotic relationships in Antarctic sponge holobionts.

Beyond these cores, the microbiomes showed host-specific diversity. Sixteen microbial phyla appear restricted to single sponge orders, with the species-rich Poecilosclerida hosting eleven unique phyla. This pattern of order-specific microbes suggests the specialisation to specific host lineages.

## Biogeographic Patterns in Antarctic Sponge Microbiomes

### Spatial Distribution and Biogeographic Trends

**Fig. 4:**
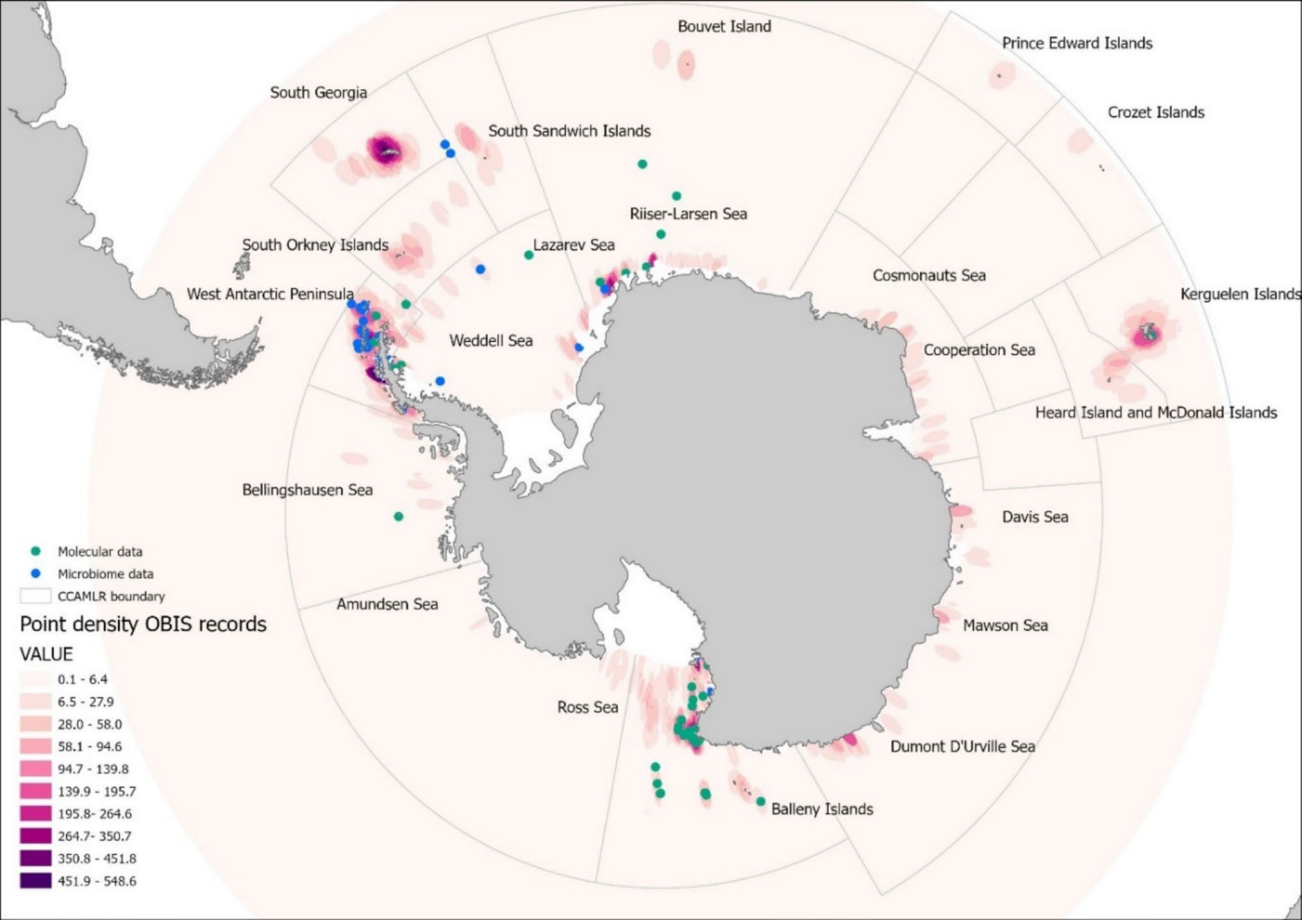
Spatial density of Antarctic sponge distribution records, microbiome studies, and DNA barcoding efforts. Point density of all distribution records of sponges from OBIS in the Antarctic, sponge microbiome studies, and DNA barcoding. Records lacking georeferencing include 298 barcoded entries (72%) and 6 microbiome study records (4%), which are not displayed on the map

Microbiomes are concentrated around the Western Antarctic Peninsula and Ross Sea, with less than 20 records from sub-Antarctic locations (Fig. 4). This spatial skew is in OBIS, with South Georgia, Lazarev Sea, and Dumont D’Urville Sea having high sampling density. This shows persistent biogeographic biases in microbiome and molecular data. Despite a fivefold increase in sponge distribution records in OBIS East Antarctica (Supplementary Fig. 2a), the open ocean between the Antarctic and sub-Antarctic islands, the Bellingshausen, Amundsen, and western Weddell seas remains poorly represented for holobionts (Supplementary Fig. 2b) [4].

Most microbiome studies are thus derived from the Antarctic Peninsula, Kerguelen Islands, and eastern Ross Sea, which constrains ecological inferences across environmental gradients. The geographic clustering of host and microbiome data (Fig. 4) highlights the need for targeted efforts to fill biogeographic and bathymetric gaps in the Antarctic sponge holobiont record.

### Bathymetric Bias in Sponge and Microbiome Sampling

**Fig. 5:**
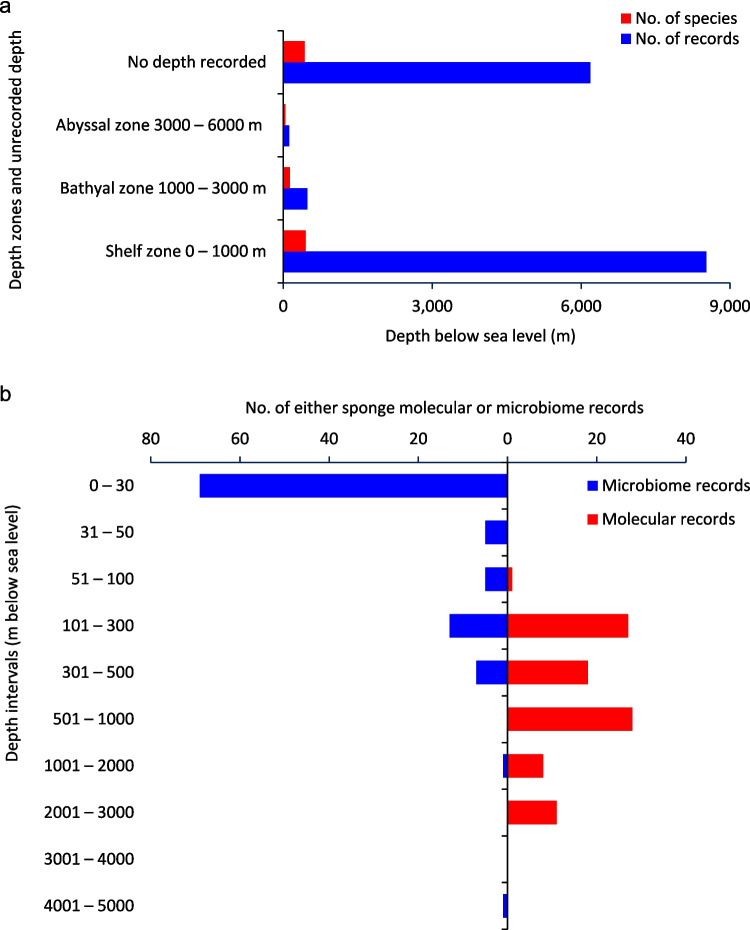
Depth distribution and record analysis of Southern Ocean sponge species and associated microbiome data. **a** Total number of OBIS species records and number of species (including subspecies/varieties) recorded across three depth zones, presented both overall and by sponge class in the Southern Ocean (OBIS, 2024). **b** Microbiome and molecular specimen records with depth information available for Antarctic sponges. Note that only 103 microbiome records (66%) held depth metadata, suggesting that the apparent concentration of records in shallower zones is likely an underrepresentation of deeper occurrences

Antarctic sponge distributions exhibit pronounced bathymetric bias, which limits the ecological interpretation of associated microbial communities. Analysis of OBIS records shows that over 90% of depth-annotated sponge occurrences originate from the continental shelf (Supplementary Table 1; Fig. 5a), despite shelf habitats comprising only a tiny fraction of the total Southern Ocean area [62, 63]. This shallow-water skew influences perceived host richness and, by extension, our understanding of holobiont diversity.

The shelf zone supports the highest documented sponge richness, dominated by Demospongiae (> 80% of records). Since 2012, shelf records have doubled, while species richness increased by only ~ 10% [4], suggesting enhanced spatial resolution rather than novel discoveries. Some classes, notably Calcarea, show apparent declines in richness, largely due to taxonomic revisions rather than ecological change [4, 14].

In contrast, bathyal zones have seen a greater than 2.5-fold increase in records and a 33% rise in species richness, particularly among Demospongiae and Hexactinellida. Abyssal sponge records have more than doubled, though total species counts remain low. Notably, only ~ 7% of all sponge records originate from depths greater than 200 m, despite such depths comprising more than 90% of the Southern Ocean area [62, 63].

This bias is mirrored in microbiome datasets: only 49% of 199 microbially sequenced sponge specimens include depth metadata, and microbial studies below 300 m remain sparse (Fig. 5b). Underrepresentation of deep-water sponges constrains our ability to resolve depth-related microbial gradients or assess holobiont responses to hydrostatic pressure, low productivity, or other depth-specific factors.

Together, these bathymetric patterns highlight persistent sampling gaps limiting host and microbial biodiversity estimates. Given the potential for deep-sea sponges to harbor distinct, stress-adapted microbiomes, systematically exploring mesopelagic and abyssal habitats is essential for a comprehensive understanding of Antarctic sponge holobionts and their microbial ecology.

### Geographic and Environmental Influences on Sponge-Microbiome Associations

The distribution of holobiont associations across Antarctic waters revealed complex patterns influenced by geography and environment. While core microbiome components remain remarkably stable across locations, 21 microbial phyla were location-specific, suggesting environmental factors shape these symbiotic communities’ variable components. These patterns emerged most clearly in studies of deep-sea and hydrothermal vent environments [8], where distinct environmental conditions have driven unique microbial assemblages.

Bathymetric gradients strongly influenced sponge and microbiome distributions. Deep-sea sponge microbiomes showed distinct characteristics from their shallow-water counterparts, typically exhibiting lower complexity but higher heterogeneity [8]. This pattern paralleled the broader distribution of sponge diversity across depth gradients, where species composition changed significantly from shelf to bathyal and abyssal zones. The sampling of only 7% of records from depths greater than 200 m suggests our understanding of deep-water sponge holobionts is incomplete.

Environmental drivers exert strong influence on Antarctic sponge microbiome structure. At hydrothermal vents, sponge-associated communities include chemosynthetic Gammaproteobacteria [55], consistent with nutrient-limited, high-pressure conditions. Such functional convergence mirrors other deep-sea chemosynthetic systems, highlighting the role of local environments in shaping microbial symbioses.

Regionally, microbiome composition correlates with water mass properties [26]. Distinct circulation and chemistry in the Ross Sea and Western Antarctic Peninsula support characteristic microbial assemblages, even as core taxa persist. This suggests a hierarchical framework of host-microbe assembly, where local factors modulate variable microbiome components without disrupting conserved associations.

Comparative studies between South American and Antarctic sponge microbiomes show clear biogeographic differentiation linked to environmental gradients [58]. Temporal surveys demonstrate that core microbiomes remain stable despite seasonal and spatial fluctuations [18, 19], reflecting strong host selection and symbiotic resilience.

Antarctic sponge holobionts tolerate environmental perturbation better than expected [60], a resilience likely stemming from evolved stability mechanisms that buffer microbial community composition across ecological transitions.

These findings underscore sponge microbiome ecological plasticity within a stable symbiotic core. While host identity defines foundational microbial partnerships, environmental conditions drive peripheral diversity, enabling adaptation across heterogeneous Antarctic habitats.

## Evolution and Adaptation of the Antarctic Sponge Holobiont

### Evolutionary History

The evolutionary trajectory of Antarctic sponge holobionts underpins their capacity to persist in extreme environments and adapt to rapid climate change. Repeated glaciation cycles and long-term geographic isolation in the Southern Ocean have selected for cold- and ice-adapted sponges and microbial partners [64–66], with variable nutrient fluxes promoting diversification [67, 68].

Molecular data from the Ross Sea indicate extensive in situ evolution (within the Southern Ocean), with diversification rates comparable to tropical systems [33]. While some sponge lineages, such as Haplosclerida and Tetractinellida, may trace their origins to Gondwanan times, others, like Poecilosclerida, exhibit more recent or complex histories [33]. Adaptation to polar conditions is evident in Tetillidae [34] and Clathrinidae [35], although taxonomic gaps persist. A comparison of microbiomes between ancient and recent sponge groups remains to be done.

Microbiome evolution is tightly linked to host phylogeny. Distinct microbial assemblages are consistently observed within sponge orders, with Poecilosclerida and Hexactinellida hosting remarkably divergent communities. The latter host is known for rapid molecular evolution [12] and maintains lineage-specific microbial groups. Calcarean sponges may have undergone recent radiations [69], though their microbiomes remain poorly characterised.

The 67% endemism in Antarctic sponges [70] and strong host-specific microbial patterns suggest long-term co-evolution between hosts and symbionts. These patterns highlight the evolutionary legacy of resilience and ecological roles of sponge holobionts associations under past change.

### Microbiome Transmission and Maintenance

Microbiome transmission and maintenance mechanisms in Antarctic sponges are unknown but may rely on the unique episodic nature of polar environments. Hosts exhibit low microbial abundance [21], dominated by specific bacterial taxa, in contrast to high microbial abundance sponges of tropical and temperate systems [18–21] (Table 1), indicating divergent strategies for symbiont selection and retention.

**Table 1:** Antarctic sponge microbiome studies reporting of both vertical and horizontal transmission

Aspect	Observations	Ref
Vertical transmission	• High host specificity of microbial communities	[19, 23, 57]
	• Consistent core microbiome components across geographically distant populations	[17]
	• Strong phylogenetic signals in microbiome composition suggesting long-term co-evolution	[23, 26, 42, 46, 57, 71–74]
	• Core prokaryotic communities that persist across generations	[19, 23, 57]
Horizontal transmission	• Approximately 10% of the sponge microbiome overlaps with surrounding seawater communities	[43]
	• Environmental acquisition appears selective, with distinct community structuring compared to seawater	[26, 75]
	• Seasonal variations suggest dynamic but controlled exchange processes	[18, 19]
	• Deep-sea sponges show distinct patterns compared to shallow-water species	[24, 26]
Mechanisms maintaining stable microbiomes	• Host-specific selection maintains beneficial symbionts	[18–21]
	• Chemical signalling networks facilitate host-microbe interactions	[76]
	• Metabolic interdependencies revealed through metagenomic studies	[8]
	• Specialised symbiotic lifestyles and nutrient cycling functions	[8]

Microbiome composition is highly host-specific and stable across spatial and environmental gradients [19, 23, 57], implying tightly regulated host-symbiont interactions. The persistence of core microbial communities across geographic distances and depth zones suggests a role for vertical transmission, while detection of local or habitat-specific taxa supports the occurrence of selective horizontal acquisition [18–21, 60, 77].

Dual transmission may underpin the resilience of Antarctic sponge holobionts under variable conditions [19, 23, 57, 60, 77]. Clarifying these processes is key for understanding microbiome assembly, predicting host responses to environmental stress [25, 58, 60], and informing conservation strategies for these ecologically essential symbioses [78].

### Gene Flow in Antarctic Sponges and Microbiome Implications

Recent studies on gene flow in Antarctic sponges have shed light on population connectivity and its implications for microbiome structure. In *Dendrilla antarctica*, there is high gene flow across the Antarctic Peninsula and South Shetland Islands, with limited substructure indicating frequent larval dispersal [36]. In contrast, *Mycale* (*Oxymycale*) *acerata* showed genetic isolation from South Georgia and the South Sandwich Islands, though admixture at the South Orkney Islands suggests this region may act as a mixing zone [36].

These species-specific connectivity patterns highlight varying dispersal capacities among Antarctic sponges, with consequences for symbiont transmission. In *D. antarctica*, host genetic distance weakly correlated with microbiome composition, while geography was a stronger predictor [25]. This suggests that environmental filtering may outweigh host genetics in shaping microbiome structure, though the contributions of vertical and horizontal transmission remain unresolved.

Given that Antarctic sponges have lecithotrophic larvae with limited dispersal, understanding how reproduction influences microbiome inheritance is key to disentangling holobiont co-evolution [25]. Additional research is needed to quantify the relative influence of host genetics, dispersal limitation, and environmental conditions on microbiome assembly across sponge taxa and habitats. This is especially relevant for taxa adapted to environments hydrothermal vents or deep-sea carnivory, where symbioses may be under strong selective pressure.

### Functional Integration

Functional integration of the sponge holobiont reflects adaptations to cold, nutrient-variable conditions. Metagenomics have identified microbial genes central to nitrogen cycling and chemoautotrophic carbon fixation, often favoring light-independent pathways suited to the polar environment [8]. These microbial functions likely support host survival in resource-limited habitats.

Distinct metabolic strategies are evident in deep-sea and hydrothermal vent sponges, where symbionts, such as chemosynthetic Gammaproteobacteria, enable persistence under narrow resource conditions [8, 24, 26, 55]. The recurrent detection of core microbial taxa across environments suggests functional redundancy and tight metabolic coupling with host processes. This stability and functional versatility highlight the ecological significance of sponge-microbe partnerships in the Southern Ocean. Understanding these integrated systems informs broader questions about microbial symbiosis, host adaptation, and resilience under environmental change.

## Response to Environmental Change

### Current Environmental Pressures

The stability of Antarctic sponge holobionts conferred by slow growth and constant environments is poor adaptations to rapid warming [79, 80]. In addition, changes in sea ice, ice shelves, and glacial systems have increase disturbance, particularly in shallow-water coastal waters where temperature fluctuations are pronounced [81–83]. These cascading impacts (Table 2) affect sponge growth and mortality [83–87], mainly among the Hexactinellida glass sponges, and change the entire sponge holobiont.

**Table 2:** Direct and indirect environmental impacts on sponges and their microbiomes

Environmental stressor	Impact on Antarctic sponges and microbiomes	Ref
Temperature increase	• Most severe impacts in shallow coastal regions • Short-term warming events trigger shifts in relative abundance of key bacterial taxa while maintaining core functions • Core microbiome shows resilience to thermal stress compared to temperate regions	[60, 77]
Ice cover change	• Alterations in seasonal ice patterns affect food availability • Changes in light penetration impact photosynthetic symbiont communities • Physical disturbance from ice scour affects recruitment	[43, 46, 83, 84]
Ocean acidification	• Primary effects on calcifying symbionts • Heterotrophic bacterial communities show more stability • Potential impacts on spicule formation and skeletal development	[8, 82]
Sedimentation	• Increased glacial melt leads to higher sediment loads • Changes in filter-feeding efficiency affect associated microbial communities • Modified particle retention impacts nutrient acquisition	[26, 83]
Food availability	• Seasonal and climate-driven changes in productivity • Adjustments in nutrient cycling within the holobiont • Shifts in microbial community composition related to resource availability	[8, 26, 67, 68, 75]

Antarctic sponge microbiomes exhibit consistent responses to a range of environmental stressors. Core microbiomes remain stable with environmental variation [18, 19] while the variable components become more variable [24, 26], a combination key for future survival. Antarctic sponge holobionts are unexpectedly resilient to environmental stress [60]. However, the effectiveness of these adaptive mechanisms under rapid environmental change remains uncertain and may vary among species and locations [82].

### Holobiont Stability and Resilience

Antarctic sponge holobionts exhibit multilayered physiological and microbial adaptations contributing to stability under Southern Ocean environmental stressors. Heat stress experiments suggest these holobionts may be more resilient than their tropical and temperate counterparts, maintaining stable core microbiomes under thermal perturbation [60]. This resilience likely reflects long-term adaptation to variable conditions in shallow Antarctic habitats, though its efficacy under accelerating climate change remains uncertain and species-specific [82].

Cellular responses include upregulation of heat shock proteins [60], lipid remodelling to preserve membrane fluidity [8], and activation of antioxidant defences [77]. Structural adaptations further support stable symbiosis under stress [23]. Microbial contributions to resilience include metabolic redundancy among symbionts, functional persistence by core taxa [18, 19], and energy acquisition via chemosynthetic pathways [8, 55]. Microbiome composition appears shaped by selective host retention of stress-tolerant strains [57], immune modulation [8], and tight metabolic integration with host functions [23, 77].

At the holobiont level, Antarctic sponges adopt reduced metabolic rates [79, 80], enhanced particle retention [83], and flexible feeding modes enabled by symbiont diversity [26]. Tissue remodelling also supports recovery from disturbance [85–87]. Together, these mechanisms illustrate the integrated strategies that support ecological persistence across Antarctic benthic environments. Elucidating such stability mechanisms will be critical for forecasting species’ vulnerability, identifying thresholds of holobiont function, and informing conservation under future climate scenarios.

## Research Gaps and Future Directions

### Current Limitations and Knowledge Gaps

Four key knowledge gaps hinder our understanding of Antarctic sponge-microbiome systems. First, methodological limitations constrain diversity estimates. High-throughput sequencing approaches show significant variation depending on the method used (Supplementary Tables 4 and 5; Table 3) [88, 89]. Diversity estimates differ across 16S rRNA gene regions [19, 42, 73, 90] (Table 4), and longer amplicons do not consistently improve taxonomic resolution [56, 57]. DNA extraction methods also introduce biases [89, 91], underscoring the need for increased use of shotgun metagenomics. Inconsistencies in metadata reporting reduce cross-study comparability [92], and variation in bioinformatic pipelines further impacts community profiling [92, 93]. Moreover, the computational demands of advanced analyses remain a barrier to broader sampling and standardisation [92].

**Table 3:** Comparison of the microbiota coverage revealed by different region-specific primers of 16S rRNA gene for the same sponge species

Sponge species	No. of bacteria phyla	No. of shared phyla	16S rRNA gene region	Primer pair	Ref
Haliclona (Rhizoniera) dancoi	6	5	V1-V2	27 F/338R	[16]
	9		V3-V4	Bact-341 F/785R	[54]
Hemigellius pilosus	7	4	V1-V2	27 F/338R	[16]
	6		V3-V4	Bact-341 F/785R	[54]
Hymeniacidon torquata	16	8	V4	515 F/806R	[43]
	10		V4	515 F/806R	[18, 57]
	14		V4-V5	515 F/926R	[18, 57]
Isodictya kerguelenensis	22	7	V4	515 F/806R	[56] [57]
	9		V4-V5	515 F/926R	
Leucetta antarctica	11	11	V4	515 F/806R	[43]
	20		Shotgun	/	[8]
Microxina sarai	8	6	V1-V2	27 F/338R	[16]
	15		V3-V4	Bact-341 F/785R	[54]
Mycale (Oxymycale) acerata	5	5	V1-V2	27 F/338R	[16]
	12		V3-V4	Bact-341 F/785R	[54]
	12		V4-V5	517 F/926R	[18]
	25		V2-V4	/	[42]
			V6-V9		
	30		V4-V5	515 F/926R	[19]

Note: *Dendrilla antarctica* is not shown in the table due to unclear number of the bacterial phyla reported [23]

**Table 4:** Recommendations for future research

Research focus	Key actions	Expected outcomes
Diversity and integrative expansion	• Use species lists and genetic records to address taxonomic biases to focus on different Antarctic sponge lineages • Apply multiple taxonomic DNA markers	Enhanced understanding of sponge genetic richness
Holistic studies of sponges and microbiomes	• Integrate sponge and microbiome studies, including metabolites • Combine culturing, amplicon sequencing, and metagenomics	Deeper insights into sponge and microbiome evolution and evolutionary factors, particularly holobiont stability
Explore microbiome and function	• Investigate symbiotic bacteria selection and quorum sensing (host specificity) • Focus on functional roles in sponge-microbe symbiosis using omics approaches • Consider earlier developmental stages of sponges	Better understanding of microbiome transmission and functions over time and space and through the development of the sponge, to identify adaptation potential across species
Expand environmental aspects	• Extend research in under-explored areas: East Antarctica, sub-Antarctic regions, sub-ice and deep-sea habitats • Establish long-term and seasonal monitoring in areas undergoing change, focusing on multiple stressor effects	Broader knowledge of sponge diversity and spatiotemporal impacts of environmental change and other stressors on sponges and their microbiomes
Standardise sampling and methodological strategies for better coordination	• Systematic sampling and sufficient replicates while measuring environmental factors • Address biases in sponge diversity data and microbiome methodologies • Share protocols and improve metadata reporting in databases • Coordinate sampling efforts and develop shared resources	Improved tracking of environmental impacts on sponge species, alongside the ability to improve efficiency and reuse of data for conservation and management of vulnerable species/communities, and cross-study comparisons

Secondly, our understanding of Antarctic sponge diversity and sponge-microbiome dynamics remains limited by spatiotemporal and taxonomic biases. Sampling is heavily skewed toward accessible regions and shallow depths, with poor representation of deep-sea, under-ice, and sub-Antarctic habitats [4, 7, 62, 63, 94]. Temporal replication is minimal [18, 19], and taxonomic coverage remains uneven. Calcarea and Homoscleromorpha are particularly underrepresented (Supplementary Table 6) [8, 43].

Thirdly, host-microbe interactions remain poorly resolved. Culture-dependent and culture-independent approaches often yield limited overlap, even for the same sponge species (e.g., Hentschel et al. [95]), complicating interpretations. Functional integration between host and symbionts is incompletely understood [8, 92], and mechanisms of microbiome transmission remain unclear [25, 76]. Data on symbiont roles in host fitness and adaptation are sparse [8, 60, 77], as are insights into co-evolutionary patterns [23, 73]. This hampers delineation of core versus variable microbiome components [19, 23, 57, 74].

Finally, as polar ecosystems undergo rapid change, the stability and resilience of sponge holobionts remain uncertain. Current data cannot assess seasonal, annual, and multi-stressor responses [18, 19, 58, 60, 81, 82]. Addressing these gaps will require coordinated sampling strategies, broader ecological coverage, and integration of environmental and multi-omic frameworks [78, 90, 93].

### Improving Methodological Standards, Integrating Approaches, and Targeting Gaps

Three priority areas require focused attention to advance understanding of Antarctic sponge-microbiome systems. First, methodological consistency is essential. Standardised DNA extraction, amplification, and sequencing protocols are needed to improve comparability and data reuse across studies [88, 89, 91]. Integrated analytical approaches, including cultivation, amplicon sequencing, metagenomics, and functional profiling, should be applied more broadly to address questions of holobiont evolution, function, and microbiome transmission [8, 92]. Robust bioinformatic pipelines are critical for efficiently managing and interpreting large-scale datasets [92, 93]. For 16S rRNA gene-based microbial diversity studies, the V4 region provides a balance of resolution and reference support and is widely used with the 515 F/806R primer pair [90]. However, primer choice and target region must align with research goals and target taxa. Studies of Southern Ocean taxa (e.g., sculpt fish and limpets [96, 97]) emphasise the need to validate primer sets for comprehensive and unbiased coverage [88, 89].

Second, improved taxonomic, temporal, and spatial coverage is needed. Underrepresented habitats such as deep-sea, under-ice, and sub-Antarctic environments should be prioritised [24, 26, 76, 98]. More frequent and longitudinal sampling across seasons will enable robust assessments of holobiont dynamics [18, 19, 78]. Taxonomic gaps, especially among Calcarea and Homoscleromorpha (Supplementary Table 6), should be addressed through targeted sampling and sequencing [8, 43].

Third, harmonised metadata standards and interoperable databases are needed to integrate morphological, molecular, and environmental data [8, 92]. Strengthening links between sponge biodiversity databases and microbial sequence repositories will enhance cross-study comparisons and facilitate global syntheses [90, 93].

Together, these improvements provide a foundation for addressing critical knowledge gaps in Antarctic sponge-microbiome ecology and enable coordinated progress toward holistic, comparative analyses across polar and non-polar systems (Table 4).

### Proposal for Examining Environmental Change Impacts

Effective assessment of Antarctic sponge holobiont responses to environmental change requires consideration of multiple interacting factors. The Western Antarctic Peninsula, experiencing rapid climate-driven shifts, provides a critical natural laboratory. Sub-Antarctic islands serve as transitional zones, while regions adjacent to retreating ice shelves, sea ice, and glaciers offer unique habitats for studying environmental impacts on holobiont structure and function.

Projected responses span environmental, ecological, and evolutionary scales. Continued warming may alter both sponge physiology and microbiome composition [60, 77], while changes in ice dynamics affect habitat stability [81–83]. Variability in primary productivity and ocean acidification may disrupt food availability and skeletal formation, respectively [67, 68, 82]. Community-level effects include altered competition [82, 99], predator–prey dynamics [86, 87], and recruitment patterns [85, 86], with the potential for emergent ecological interactions under novel conditions [81, 82].

At the evolutionary scale, selective pressures may favor more resilient symbiotic partnerships [58, 60], facilitate rapid adaptation via microbiome plasticity [18, 19], or lead to the breakdown of specialised associations [25], potentially giving rise to novel strategies of holobiont adaptation [8].

To anticipate these outcomes, future research should prioritize long-term monitoring across environmental gradients [42, 78], examine cumulative stressor effects on holobiont stability [60], evaluate lineage-specific adaptive potential [99], and elucidate mechanisms underlying resilience in persisting communities [82]. Understanding how both hosts and their microbial partners respond to accelerated change is essential for forecasting the future of Antarctic benthic ecosystems and informing conservation strategies [78].

## Conclusions

This review synthesises current knowledge of Antarctic sponge-microbiome associations, highlighting a system marked by high taxonomic diversity, ecological specificity, and adaptive capacity. Of nearly 600 sponge species reported from the Southern Ocean, around 70% endemic, fewer than 10% have microbiome data. These holobionts collectively host at least 63 bacterial, 5 archaeal, and 6 fungal phyla, yet microbiome sampling remains sparse, spatially biased, and taxonomically uneven, limiting broad ecological inferences. Antarctic sponge microbiomes differ markedly from those in lower latitudes. They are dominated by low microbial abundance communities with reduced complexity and strong host specificity, traits likely shaped by polar environmental pressures. A conserved core microbiome, dominated by Proteobacteria, Bacteroidetes, Planctomycetes, and Nitrospinae, exhibits stability across environmental gradients, suggesting long-term co-evolution and selective host filtering. Functional genomic studies highlight the essential roles of symbionts in nitrogen cycling, chemoautotrophic carbon fixation, and stress tolerance. These processes are critical for supporting holobiont survival in nutrient-poor and seasonally extreme environments. Despite these advances, critical gaps remain. Sampling biases, inconsistent analytical approaches, limited functional and phylogenomic integration, and a lack of temporal studies constrain our understanding of holobiont dynamics. Expanding geographic and host taxonomic coverage, adopting standardised protocols, and integrating culture-based, sequencing, and functional approaches are essential next steps. Long-term monitoring and multi-omics analyses will be critical to assess resilience and vulnerability under ongoing climate change. Antarctic sponge holobionts provide a powerful model for understanding microbial symbiosis, adaptation, and function in extreme environments. Elucidating their ecological roles and evolutionary trajectories is central to predicting polar ecosystem responses to environmental change and informing conservation efforts in one of Earth’s most rapidly transforming marine biomes.

## Supplementary Information

Below is the link to the electronic supplementary material.

Supplementary file1 (PDF 346 KB)

Supplementary file2 (PDF 4127 KB)

## Data Availability

No datasets were generated or analysed during the current study.
