# Supplementary material for: The Microbial Ecology of Antarctic Sponges
Source: Microb Ecol. 2025 May 17;88:44. doi: 10.1007/s00248-025-02543-y (PMC12085365; doi:10.1007/s00248-025-02543-y)
Supplement: Supplementary file 1 [file 248_2025_2543_MOESM1_ESM.pdf]

Supplementary Figures for submission to the journal Microbial Ecology

The Microbial Ecology of Antarctic Sponges

Qi Yang[1,2*], Rachel Downey[3], Jonathan S. Stark[4], Glenn J. Johnstone[4], James G. Mitchell[2]

[1]CSIRO Agriculture and Food, Urrbrae, SA 5064, Australia

[2]College of Science and Engineering, Flinders University, Bedford Park, SA 5042, Australia

[3]Fenner School of Environment & Society, Australian National University, Canberra, ACT 2601, Australia

[4]East Antarctic Monitoring Program, Australian Antarctic Division, Kingston, Tasmania 7050, Australia

*Address correspondence to: Qi Yang, q.yang@csiro.au

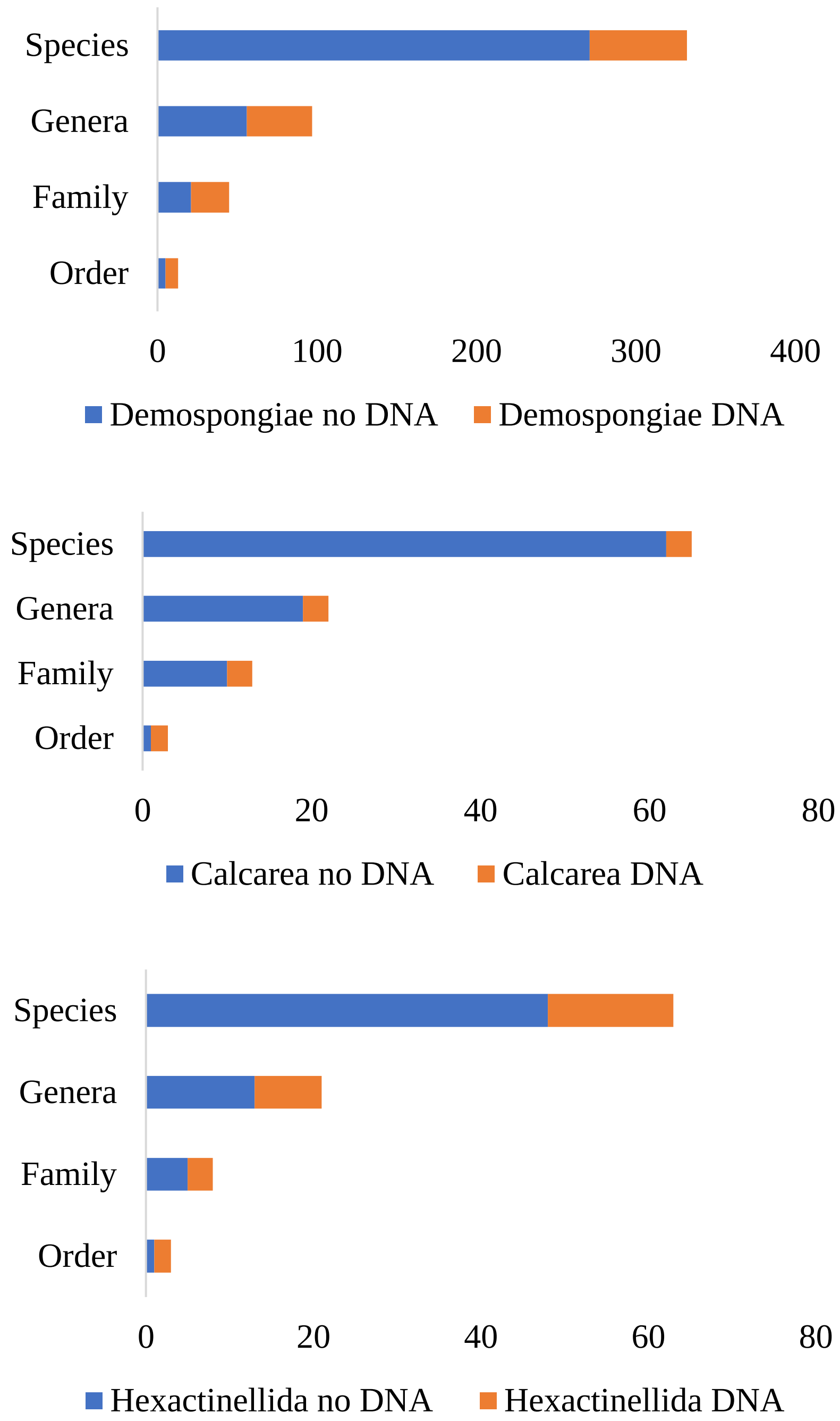

Fig. 1 The proportion of sponge species, genera, families, and order for the three sponge classes of Demospongiae (top), Calcarea (middle), and Hexactinellida (bottom) that have genetic data. The proportion of species is determined from the WoRMS-verified taxonomic lists (RAMS modified list) for Antarctic sponge species found in the CCAMLR Convention Area. The blue area combined with the orange indicates the total number of taxonomic diversity at each taxonomic level.

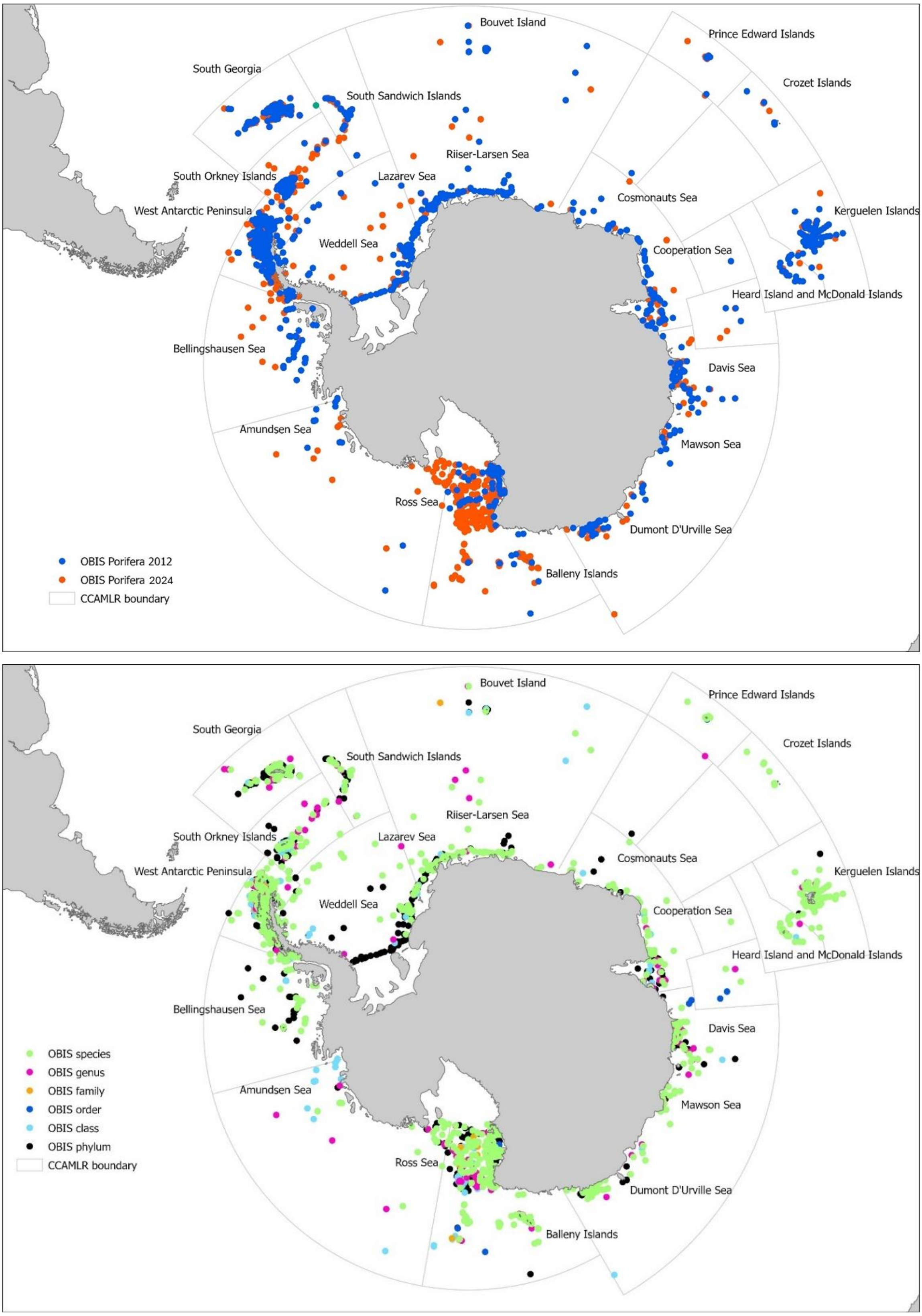

Fig. 2 Antarctic sponge richness and diversity. Map of OBIS records available in 2012 and 2024 (top). Map of the taxonomic levels of records from OBIS (2024), mapped to the CCAMLR boundary of the Southern Ocean. The highest taxonomic resolution of the record is illustrated, alongside the number of species for each CCAMLR sub-region (bottom).
