# Supplementary material for: The Microbial Ecology of Antarctic Sponges
Source: Microb Ecol. 2025 May 17;88:44. doi: 10.1007/s00248-025-02543-y (PMC12085365; doi:10.1007/s00248-025-02543-y)
Supplement: Supplementary file 2 [file 248_2025_2543_MOESM2_ESM.pdf]

# Supplementary Tables for submission to the journal Microbial Ecology

## The Microbial Ecology of Antarctic Sponges

**Qi Yang[1,2*], Rachel Downey[3], Jonathan S. Stark[4], Glenn J. Johnstone[4], James G. Mitchell[2]**

[1]CSIRO Agriculture and Food, Urrbrae, SA 5064, Australia

[2]College of Science and Engineering, Flinders University, Bedford Park, SA 5042, Australia

[3]Fenner School of Environment & Society, Australian National University, Canberra, ACT 2601, Australia

[4]East Antarctic Monitoring Program, Australian Antarctic Division, Kingston, Tasmania 7050, Australia

*Address correspondence to: Qi Yang, q.yang@csiro.au

**Table 1.** Taxonomic richness of Southern Ocean sponge classes (orders to species) based on 2023 WoRMS RAMS and OBIS data (CCAMLR boundary; OBIS values in parentheses).

| | Order | Family | Genera | Species [a] |
|---|---|---|---|---|
| **Calcarea Bowerbank, 1862** | 3 (4) | 13 (16) | 22 (24) | 65 (55) |
| **Demospongiae Sollas, 1885** | 13 (16) | 45 (56) | 95 (127) | 332 (389) |
| **Hexactinellida Schmidt, 1870** | 3 (3) | 8 (9) | 21 (30) | 63 (64) |
| **Homoscleromorpha Bergquist, 1978** | 1 (1) | 1 (2) | 1 (2) | 2 (5) |
| **Total** | 20 (24) | 67 (83) | 139 (183) | 462 (537) |

[a] includes sub-species/varieties

**Table 2.** Numbers of sequence records for each taxonomic level within each sponge class, including the total number of sequences, which also include 4 poriferan sequences only identified to phylum.

| | No. of sequences | Order | Family | Genera | (morpho)Species [a] |
|---|---|---|---|---|---|
| **Calcarea** | 12 | 2 | 3 | 3 | 3 |
| **Demospongiae** | 321 | 8 | 24 | 41 | 62 (1) |
| **Hexactinellida** | 75 | 2 | 3 | 8 | 15 |
| **Homoscleromorpha** | 0 | 0 | 0 | 0 | 0 |
| **Total** | 412 | 12 | 30 | 52 | 80 |

[a] Includes sp. if no other representative of the genus is present. Numbers in parentheses indicate species that are not verified for the Southern Ocean by WORMS (2022).

**Table 3.** Species within each class, proportioned by the number of DNA barcoding markers used.

| Class | 1 marker | 2 markers | 3 markers | 4 markers |
|---|---|---|---|---|
| **Calcarea** | 3 | 0 | 0 | 0 |
| **Demospongiae** | 45 | 8 | 8 | 1 |
| **Hexactinellida** | 0 | 1 | 4 | 10 |

Note: For mitochondrial markers/genes (257 sequences), COI (227 sequences), and nuclear markers (155 sequences).

**Table 4.** Overview of microbial community diversity of Antarctic sponges revealed by culture-dependent methods

| No. | Class | Order | Family | Genus/ Species | Microbial community diversity (Phylum level) | References |
|---|---|---|---|---|---|---|
| 1 | Hexactinellida | Lyssacinosida | Rossellidae | *Anoxycalyx joubini* | Proteobacteria, Bacteroidetes, Actinobacteria, Firmicutes | Mangano et al. 2009; Papaleo 2012; Savoca et al. 2019[1-3] |
| 2 | Hexactinellida | Lyssacinosida | Rossellidae | *Rossella nuda* * | Actinobacteria, Firmicutes | Xin et al. 2011[4] |
| 3 | Hexactinellida | Lyssacinosida | Rossellidae | *Rossella racovitzae* | Actinobacteria, Firmicutes | Xin et al. 2011[4] |
| 4 | Demospongiae | Dendroceratida | Darwinellidae | *Dendrilla* sp. | Ascomycota (Fungi) | Henríquez et al. 2014; Vaca et al. 2013[5, 6] |
| 5 | Demospongiae | Haplosclerida | Chalinidae | *Haliclona* (*Rhizoniera*) *dancoi* | Proteobacteria, Bacteroidetes, Actinobacteria, Firmicutes | Savoca et al. 2019[1] |
| 6 | Demospongiae | Haplosclerida | Chalinidae | *Haliclona* sp. | Proteobacteria, Bacteroidetes, Actinobacteria, Firmicutes | Savoca et al. 2019[1] |
| 7 | Demospongiae | Haplosclerida | Chalinidae | *Haliclona virens* | Proteobacteria, Bacteroidetes, Actinobacteria, Firmicutes | Savoca et al. 2019[1] |
| 8 | Demospongiae | Haplosclerida | Chalinidae | *Haliclona* (*Gellius*) *rudis* | Proteobacteria, Bacteroidetes, Actinobacteria, Firmicutes | Savoca et al. 2019[1] |
| 9 | Demospongiae | Haplosclerida | Niphatidae | *Haliclonissa verrucosa* | Proteobacteria, Bacteroidetes, Actinobacteria, Firmicutes | Savoca et al. 2019; Papaleo 2012; Orlandini et al. 2014[1, 2, 7] |
| 10 | Demospongiae | Haplosclerida | Niphatidae | *Hemigellius pilosus* | Proteobacteria, Bacteroidetes, Actinobacteria, Firmicutes | Savoca, S. et al. 2019 [1]<br>Mangano, S. et al. 2014 [8] |

| | | | | | | |
|---|---|---|---|---|---|---|
| 11 | Demospongiae | Haplosclerida | Phloeodictyidae | *Calyx arcuarius* | Proteobacteria, Bacteroidetes, Actinobacteria, Firmicutes | Savoca, S. et al. 2019 [1] |
| 12 | Demospongiae | Poecilosclerida | Acarnidae | *Iophon* sp. | Bacteroidota, Bacillota, Actinomycetota | Moreno-Pino, M. et al. 2020[9] |
| 13 | Demospongiae | Poecilosclerida | Coelosphaeridae | *Lissodendoryx nobilis* | Proteobacteria, Bacteroidetes, Actinobacteria, Firmicutes | Mangano et al. 2009, 2018; Savoca et al. 2019; Papaleo 2012; Orlandini et al. 2014 [1-3, 7, 10] |
| 14 | Demospongiae | Poecilosclerida | Crellidae | *Crella* sp. | Ascomycota (Fungi) | Henríquez, M. et al. 2014 [5] |
| 15 | Demospongiae | Poecilosclerida | Hymedesmiidae | *Kirkpatrickia varialosa* | Crenarchaeota (Archaea);<br>Proteobacteria, Bacteroidetes, Firmicutes | Webster, N.S. et al. 2004 [11] |
| 16 | Demospongiae | Poecilosclerida | Hymedesmiidae | *Myxodoryx hanitschi* | Proteobacteria, Bacteroidetes, Actinobacteria, Firmicutes | Savoca, S. et al. 2019; Mangano, S. et al. 2018 [1, 10] |
| 17 | Demospongiae | Poecilosclerida | Hymedesmiidae | *Phorbas glaberrimus* | Proteobacteria, Bacteroidetes, Actinobacteria, Firmicutes | Mangano, S. et al. 2018; Savoca, S. et al. 2019 [1, 10] |
| 18 | Demospongiae | Poecilosclerida | Latrunculiidae | *Latrunculia apicalis* | Crenarchaeota (Archaea);<br>Proteobacteria, Bacteroidetes, Firmicutes, Planctomyces | Webster, N.S. et al. 2004 [11] |
| 19 | Demospongiae | Poecilosclerida | Microcionidae | *Microciona* (*Clathria*)** sp. | Ascomycota (Fungi) | Henríquez, M. et al. 2014; Vaca, I. et al. 2013 [5, 6] |
| 20 | Demospongiae | Poecilosclerida | Mycalidae | *Mycale acerata* | Crenarchaeota (Archaea);<br>Proteobacteria, Bacteroidetes, Firmicutes | Webster, N.S. et al. 2004 [11] |
| 21 | Demospongiae | Poecilosclerida | Myxillidae | *Myxilla mollis* | Actinobacteria, Firmicutes | Xin Y. et al. 2011 [4] |

| | | | | | | |
|---|---|---|---|---|---|---|
| 22 | Demospongiae | Polymastiida | Polymastiidae | *Radiella antarctica* | Actinobacteria, Firmicutes | Xin Y. et al. 2011 [4] |
| 23 | Demospongiae | Polymastiida | Polymastiidae | *Sphaerotylus antarcticus* | Crenarchaeota (Archaea);<br>Proteobacteria, Bacteroidetes, Firmicutes | Webster, N.S. et al. 2004 [11] |
| 24 | Demospongiae | Poecilosclerida | Tedaniidae | *Tedania charcoti* | Proteobacteria, Bacteroidetes, Actinobacteria, Firmicutes | Savoca, S. et al. 2019 [1] |
| 25 | Demospongiae | Poecilosclerida | Tedaniidae | *Tedania* sp. | Proteobacteria, Bacteroidetes, Actinobacteria, Firmicutes<br>Ascomycota (Fungi) | Savoca et al. 2019;<br>Henríquez et al. 2014; Vaca et al. 2013 [1, 5, 6] |
| 26 | Demospongiae | Poecilosclerida | Tedaniidae | *Trachytedania spinata* | Proteobacteria, Bacteroidetes, Actinobacteria, Firmicutes | Savoca, S. et al. 2019 [1] |
| 27 | Demospongiae | Suberitida | Halichondriidae | *Hymeniacidon* sp. | Ascomycota (Fungi) | Henríquez, M. et al. 2014; Vaca, I. et al. 2013 [5, 6] |
| 28 | Demospongiae | Suberitida | Suberitidae | *Homaxinella balfourensis* | Crenarchaeota (Archaea); Proteobacteria, Bacteroidetes, Actinobacteria, Firmicutes | Xin Y. et al. 2011; Webster, N.S. et al. 2004 [4, 11] |

*The taxonomy has been newly re-identified by Senckenberg researchers as *Anoxycalyx* (*Scolymastra*) *joubini* (D. Janussen).

** Taxonomic changes mean that this is now called *Clathria* (*Microciona*) sp. (WoRMS, 2023)

**Table 5.** Overview of microbial community diversity of Antarctic sponges revealed by high-throughput sequencing

| No. | Class | Order | Family | Genus/ Species | Microbial community diversity (Phylum level *) | References |
|---|---|---|---|---|---|---|
| 1 | Calcarea | Clathrinida | Leucettidae | *Leucetta antarctica* | 20 bacterial phyla; 3 archaeal phyla: Crenarchaeota, Euryarchaeota, Thaumarchaeota; 6 fungal phyla | Moreno-Pino, M. et al. 2020[9] |
| | | | | | 11 bacterial phyla; 1 archaeal phylum: Thaumarchaeota; members in Fungi kingdom | Rodríguez-Marconi, S. et al. 2015 [12] |
| 2 | Hexactinellida | Lyssacinosida | Rossellidae | *Rossella villosa* | 5 bacterial phyla | Papale, M. et al. 2020[13] |
| 3 | Hexactinellida | Lyssacinosida | Rossellidae | *Rossella vanhoeffeni* | 13 bacterial phyla | Busch et al. 2022[14] |
| 4 | Hexactinellida | Lyssacinosida | Rossellidae | *Rossella fibulata* | 11 bacterial phyla | Busch et al. 2022[14] |
| 5 | Hexactinellida | Lyssacinosida | Rossellidae | *Anoxycalyx joubini* | 18 bacterial phyla | Busch et al. 2022[14] |
| 6 | Demospongiae | Axinellida | Axinellidae | Axinellidae sp. | 22 bacterial phyla | Busch et al. 2022[14] |
| 7 | Demospongiae | Dendroceratida | Darwinellidae | *Dendrilla antarctica* | 27 bacterial phyla; 3 archaeal phyla: Euryarchaeota, Nanoarchaeota, Thaumarchaeota ** | Sacristán-Soriano, O. et al. 2020 [15] |
| | | | | | 20 bacterial phyla; 1 archaeal phylum: Thaumarchaeota | Díez-Vives, C. et al. 2020[16] |
| 8 | Demospongiae | Haplosclerida | Chalinidae | *Haliclona* (*Rhizoniera*) *dancoi* | 6 bacterial phyla | Papale, M. et al. 2020[13] |
| | | | | | 9 bacterial phyla | Ruocco, N. et al. 2021[17] |
| 9 | Demospongiae | Haplosclerida | Chalinidae | *Haliclona* sp. | 14 bacterial phyla | Steinert, G. et al. 2019 [18] |
| 10 | Demospongiae | Haplosclerida | Chalinidae | *Haliclona* (*Gellius*) sp. | 21 bacterial phyla; 1 archaeal phylum: Thaumarchaeota; members in Fungi kingdom | Rodríguez-Marconi, S. et al. 2015 [12] |
| 11 | Demospongiae | Haplosclerida | Chalinidae | *Haliclona (Rhizoniera)* sp. | 9 bacterial phyla; 1 archaeal phylum Crenarchaeota | Cristi, A. et al. 2022[19] |
| 12 | Demospongiae | Haplosclerida | Niphatidae | *Hemigellius pilosus* | 7 bacterial phyla | Papale, M. et al. 2020 [13] |
| | | | | | 27 bacterial phyla; 3 archaeal phyla: Euryarchaeota, Nanoarchaeota, Thaumarchaeota ** | Sacristán-Soriano, O. et al. 2020 [15] |
| | | | | | 6 bacterial phyla; 1 archaeal phylum: Thaumarchaeota | Ruocco, N. et al. 2021[17] |
| 13 | Demospongiae | Haplosclerida | Niphatidae | *Microxina sarai* | 8 bacterial phyla | Papale, M. et al. 2020[13] |
| | | | | | 15 bacterial phyla; 1 archaeal phylum: Thaumarchaeota | Ruocco, N. et al. 2021[17] |
| 14 | Demospongiae | Poecilosclerida | Acarnidae | *Megaciella annectens* | 16 bacterial phyla; 1 archaeal phylum: Thaumarchaeota; members in Fungi kingdom | Rodríguez-Marconi, S. et al. 2015 [12] |

| | | | | | | |
|---|---|---|---|---|---|---|
| 15 | Demospongiae | Poecilosclerida | Cladorhizidae | *Cladorhiza* sp. | 9 bacterial phyla; 1 archaeal phylum Thaumarchaeota | Georgieva, M.N. et al. 2020[20] |
| 16 | Demospongiae | Poecilosclerida | Coelosphaeridae | *Lissodendoryx* (*Ectyodoryx*) *ramilobosa* | 5 bacterial phyla | Papale, M. et al. 2020[13] |
| 17 | Demospongiae | Poecilosclerida | Hymedesmiidae | *Kirkpatrickia varialosa* | 23 bacterial phyla; 2 archaeal phyla: Euryarchaeota, Thaumarchaeota; members in Fungi kingdom | Rodríguez-Marconi, S. et al. 2015 [12] |
| 18 | Demospongiae | Poecilosclerida | Hymedesmiidae | *Myxodoryx hanitschi* | 4 bacterial phyla: Proteobacteria, Bacteroidetes, Actinobacteria, Firmicutes | Papale, M. et al. 2020 [13] |
| 19 | Demospongiae | Poecilosclerida | Hymedesmiidae | *Phorbas* sp. | 20 bacterial phyla | Busch, K. et al. 2022[14] |
| 20 | Demospongiae | Poecilosclerida | Isodictyidae | *Isodictya bentarti* | 13 bacterial phyla | Steinert, G. et al. 2019[18] |
| 21 | Demospongiae | Poecilosclerida | Isodictyidae | *Isodictya erinacea* | 8 bacterial phyla | Papale, M. et al. 2020[13] |
| 22 | Demospongiae | Poecilosclerida | Isodictyidae | *Isodictya kerguelenensis* | 22 bacterial phyla; 1 archaea phylum: Thaumarchaeota | Rondon, R. et al. 2020[21] |
| | | | | | 9 bacterial phyla; 1 archaeal phylum Crenarchaeota | Cristi, A. et al. 2022[19] |
| 23 | Demospongiae | Poecilosclerida | Isodictyidae | *Isodictya* sp. | 13 bacterial phyla | Cárdenas, C.A. et al. 2019[22] |
| 24 | Demospongiae | Poecilosclerida | Microcionidae | *Clathria* sp. | 14 bacterial phyla; 1 archaeal phylum: Thaumarchaeota; members in Fungi kingdom | Rodríguez-Marconi, S. et al. 2015 [12] |
| 25 | Demospongiae | Poecilosclerida | Mycalidae | *Mycale* (*Aegogropila*) sp. | 4 bacterial phyla: Proteobacteria, Bacteroidetes, Actinobacteria, Firmicutes | Papale, M. et al. 2020[13] |
| 26 | Demospongiae | Poecilosclerida | Mycalidae | *Mycale* (*Oxymycale*) *acerata* | 12 bacterial phyla | Cárdenas, C.A. et al. 2019[22] |
| | | | | | 25 bacterial phyla | Cárdenas, C.A. et al. 2018[23] |
| | | | | | 27 bacterial phyla; 3 archaeal phyla: Euryarchaeota, Nanoarchaeota, Thaumarchaeota ** | Sacristán-Soriano, O. et al. 2020 [15] |
| | | | | | 5 bacterial phyla | Papale, M. et al. 2020[13] |
| | | | | | 12 bacterial phyla | Ruocco, N. et al. 2021[17] |
| | | | | | 31 bacterial phyla; 3 archaeal phyla: Crenarchaeota; Halobacterota; Nanoarchaeota | Happel, L. et al. 2022[24] |
| 27 | Demospongiae | Poecilosclerida | Myxillidae | *Myxilla* (*Burtonanchora*) sp. | 16 bacterial phyla; 1 archaeal phylum: Thaumarchaeota; members in Fungi kingdom | Rodríguez-Marconi, S. et al. 2015 [12] |
| | | | | | 20 bacterial phyla; 3 archaeal phyla: Crenarchaeota, Euryarchaeota, Thaumarchaeota; 7 fungal phyla | Moreno-Pino, M. et al. 2020[9] |

| | | | | | | |
|---|---|---|---|---|---|---|
| 28 | Demospongiae | Poecilosclerida | Tedaniidae | *Tedania* (*Tedaniopsis*) *oxeata* | 6 bacterial phyla | Papale, M. et al. 2020[13] |
| 29 | Demospongiae | Poecilosclerida | Tedaniidae | *Tedania* (*Tedaniopsis*) *wellsae* | 12 bacterial phyla | Cárdenas, C.A. et al. 2019[22] |
| 30 | Demospongiae | Polymastiida | Polymastiidae | *Sphaerotylus antarcticus* | 27 bacterial phyla; 3 archaeal phyla: Euryarchaeota, Nanoarchaeota, Thaumarchaeota ** | Sacristán-Soriano, O. et al. 2020 [15] |
| 31 | Demospongiae | Polymastiida | Polymastiidae | *Tentorium papillatum* | 25 bacterial phyla | Busch, K. et al. 2022[14] |
| 32 | Demospongiae | Suberitida | Halichondriidae | *Hymeniacidon torquata* | 16 bacterial phyla, 1 archaeal phylum: Thaumarchaeota; members in Fungi kingdom | Rodríguez-Marconi, S. et al. 2015 [12] |
| | | | | | 14 bacterial phyla | Cárdenas, C.A. et al. 2019[22] |
| | | | | | 10 bacterial phyla; 1 archaeal phylum Crenarchaeota | Cristi, A. et al. 2022 |
| 33 | Demospongiae | Suberitida | Suberitidae | *Homaxinella balfourensis* | 14 bacterial phyla | Steinert, G. et al. 2019 [18] |
| 34 | Demospongiae | Tetractinellida | Tetillidae | *Antarctotetilla leptoderma* | 21 bacterial phyla | Steinert, G. et al. 2019[18] |
| 35 | Demospongiae | Tetractinellida | Tetillidae | *Cinachyra antarctica* | 8 bacterial phyla | Papale, M. et al. 2020[13] |

*Phyla are listed if the number is less than five.

** The number of the bacterial phyla are revealed from all the sponge species in the study.

**Table 6.** Prioritisation of Antarctic sponge taxa for microbiome, ecological, and evolutionary research

| Host Class/ Order/ Species (or Genus/Family) | Host Importance | Microbiome Importance | Rationale for Prioritisation | Priority | Key References | Availability | | |
|---|---|---|---|---|---|---|---|---|
| | | | | | | Sponge | Seq | Micro |
| **High-level of available data: Identified as having large numbers of records, specimens, and sequences from several publications** | | | | | | | | |
| Demospongiae Dendroceratida ***Dendrilla antarctica*** | Widespread in shallow waters, gene flow known, prey–predator dynamics, and unusual seasonal metabolic profile | Diverse with some unique features as well as bioactive compounds | Chemical ecology, gene flow & warming sensitivity | ★★ | [25-29] | | | |
| Demospongiae Tetractinellida ***Antarctotetilla leptoderma*** | Widespread, known coloniser, from a taxonomically complex and genetically cryptic group | High levels of diversity with some unique features | Speciation, disturbance & phylogeography | ★★ | [18, 30, 31] | | | |
| Demospongiae Poecilosclerida ***Mycale (Oxymycale) acerata*** | Widespread, resilient, fast-growing known coloniser, gene flow known, and with strong metabolic seasonal changes | Spatiotemporally stable microbiome which enable it to be used as a microbiome model | Microbiome structure, gene flow, colonisation & climate resilience | ★★★ | [11, 26, 32, 33] | | | |
| Hexactinellida Lyssacinosida ***Anoxycalyx (Scolymastra) joubini*** | Long-lived, structural sponge, which is widespread on the Antarctic shelf but globally limited genus | Moderately diverse microbial associations | Growth, disturbance response, reproduction & habitat structure | ★★ | [14, 26, 31, 34] | | | |
| Hexactinellida Lyssacinosida ***Rossella* spp.** | Habitat-forming hexactinellid with a likely highly endemic species complex | Moderate and distinct microbiomes | Cryptic speciation, benthic structure, disturbance, reproduction, eurybathy & evolution | ★★★ | [13, 14, 26, 31, 35] | | | |
| **Intermediate-level of available data: Identified as having moderate numbers of records, specimens, and sequences from several publications** | | | | | | | | |
| Demospongiae Poecilosclerida **Hymedesmiidae spp. *(Kirkpatrickia, Phorbas)*** | Taxonomically diverse and with some host species widespread in either shallower shelf or deeper waters | Moderate to high levels of diversity with some potentially unique features | Cryptic speciation, eurybathy & potential for uncovering evolutionary relationships | ★★ | [10, 12, 14, 29, 36, 37] | | | |
| Demospongiae Poecilosclerida ***Iophon spp.*** | Moderately diverse, several species potentially regionally restricted | Exhibits high microbial diversity and unique functional guilds | Microbiome function & ecological role | ★★ | [9, 28, 29] | | | |
| Demospongiae Poecilosclerida ***Isodictya spp.*** | Widespread hosts, taxonomically diverse, and with a strong seasonal metabolic profile | Moderate to high levels of diversity with some potentially unique features, and microbiome | Evolutionary divergence, species complexity & environmental stress studies | ★★ | [21, 26, 29, 33] | | | |

| | | | | | |
|---|---|---|---|---|---|
| | | responds dynamically to temperature changes | | | |
| Demospongiae Poecilosclerida **Microcionidae spp. *(Artemisina, Clathria)*** | Taxonomically diverse and coloniser of ice-scoured substrate | Moderately diverse microbial associations from one species | Unexplored diversity & response to disturbance | ★★ | [12, 19, 28, 29] |
| Demospongiae Poecilosclerida ***Myxilla spp.*** | Taxonomically diverse, known coloniser, with some host species widespread | Moderate to high levels of microbial diversity found in several species | Microbial biogeography & response to stress and disturbance | ★★ | [9, 12, 29, 37] |
| Demospongiae Poecilosclerida ***Tedania spp.*** | Relatively widespread species, moderately diverse, but likely understudied taxon | Specialized microbial communities found despite low to moderate levels of diversity | Unexplored diversity & functional potential of rare taxa | ★★ | [1, 13, 28, 35] |
| Demospongiae Polymastiida ***Sphaerotylus antarcticus*** | Widespread, potential microhabitat provider, with a strong seasonal metabolic profile | Understudied but has a microbiome which needs more research | Habitat-forming & potential indicator of benthic change | ★★ | [11, 15] |
| Demospongiae Polymastiida ***Tentorium papillatum*** | Widespread but with limited global diversity | Diverse with some unique features | Insight into sponge evolution & endemism | ★★ | [14, 35] |
| Demospongiae Suberitida ***Homaxinella balfourensis*** | Widespread coloniser of ice-scoured environments with a strong seasonal metabolic profile | Understudied microbiome that could indicate novelty in this pioneer | Monitoring environmental stress & disturbance response | ★★ | [11, 18, 38, 39] |
| Demospongiae Tetractinellida ***Cinachyra spp.*** | Widespread, known coloniser, from a taxonomically complex and genetically cryptic group | Moderate diversity with some unique features | Microbiome function of a slow-growing organism, evolution & disturbance | ★★ | [13, 26, 30, 31, 35] |
| **Low-level of available data: Identified as having either limited records, specimens and/or sequences from several publications** | | | | | |
| Calcarea/ Clathrinida **Clathrinida spp. (Clathrinidae, Dendyidae, Leucascididae, Leucettidae)** | Taxonomically diverse, several endemic genera, and understudied apart from *Leucetta antarctica* | Unique and diverse features found which could be used to track environmental change | Ocean acidification, reproduction & microbiome response important to monitor | ★★★ | [11, 12, 26, 40, 41] |
| Calcarea Leucosolenida **Leucosolenida spp. *(Achramorpha, Grantia, Jenkina, Leucandra, Megapogon, Sycon)*** | Taxonomically diverse, several endemic genera, and understudied | Unique and diverse features found which could be used to track environmental change | Ocean acidification, reproduction & microbiome response important to monitor | ★★★ | [11, 26, 40, 41] |

| | | | | | | | | |
|---|---|---|---|---|---|---|---|---|
| Demospongiae Axinellida **Axinellidae spp.** ***(Axinella, Phakellia)*** | Taxonomically diverse under-studied family | Moderate levels of diversity but research is limited | Potential indicator of environmental gradients on deep shelf environments & phylogenetic divergence and glacial refugia | ★ | [42] | Amber | Amber | Amber |
| Demospongiae Haplosclerida ***Haliclona spp.*** | Taxonomic diversity with some host species widespread at shallower depths | Moderate level diversity and some unique elements, with a potential for symbiotic specialization | Symbiosis evolution & unique microbial associations | ★★ | [12, 13, 18, 26, 29, 31, 37] | Green | Amber | Amber |
| Demospongiae Poecilosclerida **Cladorhizidae spp.** | Carnivorous, taxonomically diverse, and occupies a deep-sea niche | Unusual bacteria have been found which support carnivorous sponges | Trophic ecology & deep-sea specialisation | ★★ | [20, 26, 43] | Green | Green | Red |
| Demospongiae Suberitida ***Stylocordyla chupachups*** | Forms dense populations correlated with areas of high productivity and has a strong seasonal metabolic profile | No known microbiome studies, but could be important in Suberitida evolution | Trophic innovation, reproduction & colonisation | ★★ | [44] | Green | Amber | Red |
| Hexactinellida Amphidiscosida ***Hyalonema*** **spp.** | Taxonomically diverse, several endemic species, and locally abundant in some deep-sea environments | Microbiome data sparse globally; potential for symbiotic archaea or chemosymbionts | Deep benthic adaptation & evolution | ★ | [45] | Amber | Red | Red |
| Hexactinellida Lyssacinosida **Lyssacinosida spp. (Euplectellidae and other Rossellidae -** ***Bathydorus, Caulphacus)*** | Structurally important in deep-sea habitats, taxonomcally diverse with many endemic species | Microbiome data sparse globally; potential for symbiotic archaea or chemosymbionts | Deep benthic adaptation & evolution | ★★ | [26] | Green | Amber | Red |
| Hexactinellida Sceptrulophora **Sceptrulophora spp.** ***(Chonelasma, Lonchiphora)*** | Less well-known group of deep-sea hexactinellids, with an increasing understanding of taxonomic diversity | Microbiome data sparse globally; potential for symbiotic archaea or chemosymbionts | Deep benthic adaptation & evolution | ★ | [26, 46] | Amber | Amber | Red |
| Homoscleromorpha Homosclerophorida **Plakinidae spp.** | Rare around Antarctica with few species found | Known for diverse symbiotic microbes elsewhere but Antarctic forms understudied | Polar adaptation & basal lineage in sponge phylogeny | ★★ | Costa et al. (2022)[47] | Amber | Green | Red |

Note: These hosts were selected based on their ecological importance, evolutionary interest, or unique and understudied microbiomes. A three-star priority system indicates which groups should be studied first. Data availability for each taxon is shown using colour codes: green (high), amber (moderate), and red (low), based on existing records from OBIS and genetic databases (BOLD, GenBank, EMBL-EBI, SBD).

## Reference


1. Savoca S, Lo Giudice A, Papale M, Mangano S, Caruso C, Spanò N, Michaud L, Rizzo C (2019) Antarctic sponges from the Terra Nova Bay (Ross Sea) host a diversified bacterial community. Scientific Reports 9: 16135. doi: 10.1038/s41598-019-52491-0
2. Papaleo MC, Fondi M, Maida I, Perrin E, Lo Giudice A, Michaud L, Mangano S, Bartolucci G, Romoli R, Fani R (2012) Sponge-associated microbial Antarctic communities exhibiting antimicrobial activity against Burkholderia cepacia complex bacteria. Biotechnology Advances 30: 272-293. doi: https://doi.org/10.1016/j.biotechadv.2011.06.011
3. Mangano S, Michaud L, Caruso C, Brilli M, Bruni V, Fani R, Lo Giudice A (2009) Antagonistic interactions between psychrotrophic cultivable bacteria isolated from Antarctic sponges: a preliminary analysis. Research in Microbiology 160: 27-37. doi: https://doi.org/10.1016/j.resmic.2008.09.013
4. Xin Y, Kanagasabhapathy M, Janussen D, Xue S, Zhang W (2011) Phylogenetic diversity of Gram-positive bacteria cultured from Antarctic deep-sea sponges. Polar Biology 34: 1501-1512. doi: 10.1007/s00300-011-1009-y
5. Henríquez M, Vergara K, Norambuena J, Beiza A, Maza F, Ubilla P, Araya I, Chávez R, San-Martín A, Darias J, Darias MJ, Vaca I (2014) Diversity of cultivable fungi associated with Antarctic marine sponges and screening for their antimicrobial, antitumoral and antioxidant potential. World Journal of Microbiology and Biotechnology 30: 65-76. doi: 10.1007/s11274-013-1418-x
6. Vaca I, Faúndez C, Maza F, Paillavil B, Hernández V, Acosta F, Levicán G, Martínez C, Chávez R (2013) Cultivable psychrotolerant yeasts associated with Antarctic marine sponges. World Journal of Microbiology and Biotechnology 29: 183-189. doi: 10.1007/s11274-012-1159-2
7. Orlandini V, Maida I, Fondi M, Perrin E, Papaleo MC, Bosi E, de Pascale D, Tutino ML, Michaud L, Lo Giudice A, Fani R (2014) Genomic analysis of three sponge-associated Arthrobacter Antarctic strains, inhibiting the growth of Burkholderia cepacia complex bacteria by synthesizing volatile organic compounds. Microbiological Research 169: 593-601. doi: https://doi.org/10.1016/j.micres.2013.09.018
8. Mangano S, Michaud L, Caruso C, Lo Giudice A (2014) Metal and antibiotic resistance in psychrotrophic bacteria associated with the Antarctic sponge Hemigellius pilosus (Kirkpatrick, 1907). Polar Biology 37: 227-235. doi: 10.1007/s00300-013-1426-1
9. Moreno-Pino M, Cristi A, Gillooly JF, Trefault N (2020) Characterizing the microbiomes of Antarctic sponges: a functional metagenomic approach. Scientific reports 10: 1-12.
10. Mangano S, Caruso C, Michaud L, Lo Giudice A (2018) First evidence of quorum sensing activity in bacteria associated with Antarctic sponges. Polar Biology 41: 1435-1445. doi: 10.1007/s00300-018-2296-3
11. Webster N, Negri A, Munro M, Battershill C (2004) Diverse microbial communities inhabit Antarctic sponges. Environ Microbiol 6: 288-300.
12. Rodríguez-Marconi S, De la Iglesia R, Díez B, Fonseca CA, Hajdu E, Trefault N (2015) Characterization of bacterial, archaeal and eukaryote symbionts from Antarctic sponges reveals a high diversity at a three-domain level and a particular signature for this ecosystem. PLoS One 10: e0138837.

13. Papale M, Rizzo C, Fani R, Bertolino M, Costa G, Paytuví-Gallart A, Schiaparelli S, Michaud L, Azzaro M, Lo Giudice A (2020) Exploring the diversity and metabolic profiles of bacterial communities associated with Antarctic sponges (Terra Nova Bay, Ross Sea). Frontiers in Ecology and Evolution: 268.
14. Busch K, Slaby BM, Bach W, Boetius A, Clefsen I, Colaço A, Creemers M, Cristobo J, Federwisch L, Franke A, Gavriilidou A, Hethke A, Kenchington E, Mienis F, Mills S, Riesgo A, Ríos P, Roberts EM, Sipkema D, Pita L, Schupp PJ, Xavier J, Rapp HT, Hentschel U (2022) Biodiversity, environmental drivers, and sustainability of the global deep-sea sponge microbiome. Nat Commun 13: 5160. doi: 10.1038/s41467-022-32684-4
15. Sacristán-Soriano O, Pérez Criado N, Avila C (2020) Host species determines symbiotic community composition in Antarctic sponges (Porifera: Demospongiae). Frontiers in Marine Science 7: 474.
16. Díez-Vives C, Taboada S, Leiva C, Busch K, Hentschel U, Riesgo A (2020) On the way to specificity-Microbiome reflects sponge genetic cluster primarily in highly structured populations. Molecular ecology 29: 4412-4427.
17. Ruocco N, Esposito R, Bertolino M, Zazo G, Sonnessa M, Andreani F, Coppola D, Giordano D, Nuzzo G, Lauritano C (2021) A metataxonomic approach reveals diversified bacterial communities in antarctic sponges. Marine drugs 19: 173.
18. Steinert G, Wemheuer B, Janussen D, Erpenbeck D, Daniel R, Simon M, Brinkhoff T, Schupp PJ (2019) Prokaryotic diversity and community patterns in Antarctic continental shelf sponges. Frontiers in Marine Science 6: 297.
19. Cristi A, Parada-Pozo G, Morales-Vicencio F, Cárdenas CA, Trefault N (2022) Variability in Host Specificity and Functional Potential of Antarctic Sponge-Associated Bacterial Communities. Frontiers in Microbiology: 4024.
20. Georgieva MN, Taboada S, Riesgo A, Díez-Vives C, De Leo FC, Jeffreys RM, Copley JT, Little CT, Ríos P, Cristobo J (2020) Evidence of vent-adaptation in sponges living at the periphery of hydrothermal vent environments: ecological and evolutionary implications. Frontiers in microbiology: 1636.
21. Rondon R, González-Aravena M, Font A, Osorio M, Cárdenas CA (2020) Effects of Climate Change Stressors on the Prokaryotic Communities of the Antarctic Sponge Isodictya kerguelenensis. Frontiers in Ecology and Evolution: 262.
22. Cárdenas CA, Font A, Steinert G, Rondon R, González-Aravena M (2019) Temporal stability of bacterial communities in Antarctic sponges. Frontiers in microbiology: 2699.
23. Cárdenas CA, González-Aravena M, Font A, Hestetun JT, Hajdu E, Trefault N, Malmberg M, Bongcam-Rudloff E (2018) High similarity in the microbiota of cold-water sponges of the Genus Mycale from two different geographical areas. PeerJ 6: e4935.
24. Happel L, Rondon R, Font A, González-Aravena M, Cárdenas CA (2022) Stability of the Microbiome of the Sponge Mycale (Oxymycale) acerata in the Western Antarctic Peninsula. Front Microbiol 13: 827863. doi: 10.3389/fmicb.2022.827863
25. McClintock JB, Amsler CD, Baker BJ, van Soest RWM (2005) Ecology of Antarctic marine sponges: an overview. Integrative and Comparative Biology 45: 359-368.

26. Janussen D, Downey RV (2014) Biogeographic atlas of the Southern Ocean. In: De Broyer, C, Koubbi, P, Griffiths, HJ, Raymond, B, Udekem d'Acoz, Cd (eds.) Porifera. Cambridge: Scientific Committee on Antarctic Research., pp. 94-102
27. Leiva C, Taboada S, Kenny NJ, Combosch D, Giribet G, Jombart T, Riesgo A (2019) Population substructure and signals of divergent adaptive selection despite admixture in the sponge Dendrilla antarctica from shallow waters surrounding the Antarctic Peninsula. Molecular Ecology 28: 3151-3170.
28. Freitas MAMd, Cunha-Ferreira IC, Leal CV, Fernandez JCC, Omachi CY, Campos LS, Masi BP, Krüger RH, Hajdu E, Thompson CC, Thompson FL (2023) Microbiome diversity from sponges biogeographically distributed between South America and Antarctica. Science of The Total Environment 879: 163256. doi: https://doi.org/10.1016/j.scitotenv.2023.163256
29. Moreno-Pino M, Manrique-de-la-Cuba MF, López-Rodríguez M, Parada-Pozo G, Rodríguez-Marconi S, Ribeiro CG, Flores-Herrera P, Guajardo M, Trefault N (2024) Unveiling microbial guilds and symbiotic relationships in Antarctic sponge microbiomes. Scientific Reports 14: 6371. doi: 10.1038/s41598-024-56480-w
30. Carella M, Agell G, Cárdenas P, Uriz MJ (2016) Phylogenetic reassessment of Antarctic Tetillidae (Demospongiae, Tetractinellida) Reveals New Genera and Genetic Similarity among Morphologically Distinct Species. PLoS ONE 11: e0160718.
31. Dayton PK, Jarrell SC, Kim S, Ed Parnell P, Thrush SF, Hammerstrom K, Leichter JJ (2019) Benthic responses to an Antarctic regime shift: food particle size and recruitment biology. Ecological Applications 29: e01823.
32. Barnes DK, Sands CJ, Hogg OT, Robinson BJ, Downey RV, Smith JA (2016) Biodiversity signature of the last glacial maximum at South Georgia, Southern Ocean. Journal of Biogeography 43: 2391-2399.
33. Cárdenas CA, González-Aravena M, Font A, Hestetun JT, Hajdu E, Trefault N, Malmberg M, Bongcam-Rudloff E (2018) High similarity in the microbiota of cold-water sponges of the Genus Mycale from two different geographical areas. PeerJ 6.
34. Dayton PK, Kim S, Jarrell SC, Oliver JS, Hammerstrom K, Fisher JL, O'Connor K, Barber JS, Robilliard G, Barry J (2013) Recruitment, growth and mortality of an Antarctic hexactinellid sponge, Anoxycalyx joubini. PloS one 8: e56939.
35. Vargas S, Kelly M, Schnabel K, Mills S, Bowden D, Wörheide G (2015) Diversity in a Cold Hot-Spot: DNA-Barcoding Reveals Patterns of Evolution among Antarctic Demosponges (Class Demospongiae, Phylum Porifera). PLoS ONE 10: e0127573.
36. Koutsouveli V, Taboada S, Moles J, Cristobo J, Ríos P, Bertran A, Solà J, Avila C, Riesgo A (2018) Insights into the reproduction of some Antarctic dendroceratid, poecilosclerid, and haplosclerid demosponges. PLoS One 13: e0192267.
37. Manrique-de-la-Cuba MF, Parada-Pozo G, Rodríguez-Marconi S, López-Rodríguez MR, Abades S, Trefault N (2024) Evidence of habitat specificity in sponge microbiomes from Antarctica. Environmental Microbiome 19: 100. doi: 10.1186/s40793-024-00648-4
38. Dayton PK (1989) Interdecadal variation in an Antarctic sponge and its predators from oceanographic climate shifts. Science 245: 1484-1486.

39. McClintock JB, Baker BJ, Slattery M, Hamann M, Kopitzke R, Heine J (1994) Chemotactic tube-foot responses of a spongivorous sea star Perknaster fuscus to organic extracts from antarctic sponges. Journal of Chemical Ecology 20: 859-870.
40. McClintock JB, Amsler CD, Baker BJ, Van Soest RW (2005) Ecology of Antarctic marine sponges: an overview. Integr Comp Biol 45: 359-368.
41. Riesgo A, Cavalcanti FF, Kenny NJ, Ríos P, Cristobo J, Lanna E (2018) Integrative systematics of clathrinid sponges: morphological, reproductive and phylogenetic characterisation of a new species of Leucetta from Antarctica (Porifera, Calcarea, Calcinea) with notes on the occurrence of flagellated sperm. Invertebrate Systematics 32: 827-841.
42. Díez-Vives C, Koutsouveli V, Conejero M, Riesgo A (2022) Global patterns in symbiont selection and transmission strategies in sponges. Frontiers in Ecology and Evolution Volume 10 - 2022. doi: 10.3389/fevo.2022.1015592
43. Vacelet J, Boury-Esnault N, Fiala-Medioni A, Fisher CR (1995) A methanotrophic carnivorous sponge. Nature 377: 296.
44. Carella M, Agell G, Uriz MJ (2019) Asexual reproduction and heterozygote selection in an Antarctic demosponge (Stylocordyla chupachus, Suberitida). Polar Biology 42: 475-483.
45. Beaulieu SE (2001) Life on glass houses: sponge stalk communities in the deep sea. Marine Biology 138: 803-817. doi: 10.1007/s002270000500
46. Janussen D, Tendal OS (2007) Diversity and distribution of Porifera in the bathyal and abyssal Weddell Sea and adjacent areas. Deep-Sea Research II 54: 1864-1875.
47. Costa G, Bavestrello G, Canese S, Canessa M, Mazzoli C, Montagna P, Puce S, Schiaparelli S, Bertolino M (2022) Sponges associated with stylasterid thanatocoenosis (Cnidaria, Hydrozoa) from the deep Ross Sea (Southern Ocean). Polar Biology 45: 703-718. doi: 10.1007/s00300-022-03023-6
