# Supplementary material for: The Microbial Ecology of Antarctic Sponges
Source: Microb Ecol. 2025 May 17;88(1):44. doi: 10.1007/s00248-025-02543-y (PMC12085365; doi:10.1007/s00248-025-02543-y)
Supplement: Supplementary file 2 — Supplementary file2 (PDF 4127 KB) [file 248_2025_2543_MOESM2_ESM.pdf]

Supplementary Tables for submission to the journal Microbial Ecology

The Microbial Ecology of Antarctic Sponges

Qi Yang[1,2*], Rachel Downey[3], Jonathan S. Stark[4], Glenn J. Johnstone[4], James G. Mitchell[2]

[1]CSIRO Agriculture and Food, Urrbrae, SA 5064, Australia

[2]College of Science and Engineering, Flinders University, Bedford Park, SA 5042, Australia

[3]Fenner School of Environment & Society, Australian National University, Canberra, ACT 2601, Australia

[4]East Antarctic Monitoring Program, Australian Antarctic Division, Kingston, Tasmania 7050, Australia

*Address correspondence to: Qi Yang, q.yang@csiro.au

Table 1. Taxonomic richness of Southern Ocean sponge classes (orders to species) based on 2023 WoRMS RAMS and OBIS data (CCAMLR boundary; OBIS values in parentheses).

	Order	Family	Genera	Species [a]
**Calcarea Bowerbank, 1862**	3 (4)	13 (16)	22 (24)	65 (55)
**Demospongiae Sollas, 1885**	13 (16)	45 (56)	95 (127)	332 (389)
**Hexactinellida Schmidt, 1870**	3 (3)	8 (9)	21 (30)	63 (64)
**Homoscleromorpha Bergquist, 1978**	1 (1)	1 (2)	1 (2)	2 (5)
**Total**	20 (24)	67 (83)	139 (183)	462 (537)

[a] includes sub-species/varieties

Table 2. Numbers of sequence records for each taxonomic level within each sponge class, including the total number of sequences, which also include 4 poriferan sequences only identified to phylum.

	No. of sequences	Order	Family	Genera	(morpho)Species [a]
**Calcarea**	12	2	3	3	3
**Demospongiae**	321	8	24	41	62 (1)
**Hexactinellida**	75	2	3	8	15
**Homoscleromorpha**	0	0	0	0	0
**Total**	412	12	30	52	80

[a] Includes sp. if no other representative of the genus is present. Numbers in parentheses indicate species that are not verified for the Southern Ocean by WORMS (2022).

Table 3. Species within each class, proportioned by the number of DNA barcoding markers used.

Class	1 marker	2 markers	3 markers	4 markers
**Calcarea**	3	0	0	0
**Demospongiae**	45	8	8	1
**Hexactinellida**	0	1	4	10

Note: For mitochondrial markers/genes (257 sequences), COI (227 sequences), and nuclear markers (155 sequences).

Table 4. Overview of microbial community diversity of Antarctic sponges revealed by culture-dependent methods

No.	Class	Order	Family	Genus/ Species	Microbial community diversity (Phylum level)	References
1	Hexactinellida	Lyssacinosida	Rossellidae	*Anoxycalyx joubini*	Proteobacteria, Bacteroidetes, Actinobacteria, Firmicutes	Mangano et al. 2009; Papaleo 2012; Savoca et al. 2019[1-3]
2	Hexactinellida	Lyssacinosida	Rossellidae	*Rossella nuda* *	Actinobacteria, Firmicutes	Xin et al. 2011[4]
3	Hexactinellida	Lyssacinosida	Rossellidae	*Rossella racovitzae*	Actinobacteria, Firmicutes	Xin et al. 2011[4]
4	Demospongiae	Dendroceratida	Darwinellidae	*Dendrilla* sp.	Ascomycota (Fungi)	Henríquez et al. 2014; Vaca et al. 2013[5, 6]
5	Demospongiae	Haplosclerida	Chalinidae	*Haliclona* (*Rhizoniera*) *dancoi*	Proteobacteria, Bacteroidetes, Actinobacteria, Firmicutes	Savoca et al. 2019[1]
6	Demospongiae	Haplosclerida	Chalinidae	*Haliclona* sp.	Proteobacteria, Bacteroidetes, Actinobacteria, Firmicutes	Savoca et al. 2019[1]
7	Demospongiae	Haplosclerida	Chalinidae	*Haliclona virens*	Proteobacteria, Bacteroidetes, Actinobacteria, Firmicutes	Savoca et al. 2019[1]
8	Demospongiae	Haplosclerida	Chalinidae	*Haliclona* (*Gellius*) *rudis*	Proteobacteria, Bacteroidetes, Actinobacteria, Firmicutes	Savoca et al. 2019[1]
9	Demospongiae	Haplosclerida	Niphatidae	*Haliclonissa verrucosa*	Proteobacteria, Bacteroidetes, Actinobacteria, Firmicutes	Savoca et al. 2019; Papaleo 2012; Orlandini et al. 2014[1, 2, 7]
10	Demospongiae	Haplosclerida	Niphatidae	*Hemigellius pilosus*	Proteobacteria, Bacteroidetes, Actinobacteria, Firmicutes	Savoca, S. et al. 2019 [1]<br>Mangano, S. et al. 2014 [8]

11	Demospongiae	Haplosclerida	Phloeodictyidae	*Calyx arcuarius*	Proteobacteria, Bacteroidetes, Actinobacteria, Firmicutes	Savoca, S. et al. 2019 [1]
12	Demospongiae	Poecilosclerida	Acarnidae	*Iophon* sp.	Bacteroidota, Bacillota, Actinomycetota	Moreno-Pino, M. et al. 2020[9]
13	Demospongiae	Poecilosclerida	Coelosphaeridae	*Lissodendoryx nobilis*	Proteobacteria, Bacteroidetes, Actinobacteria, Firmicutes	Mangano et al. 2009, 2018; Savoca et al. 2019; Papaleo 2012; Orlandini et al. 2014 [1-3, 7, 10]
14	Demospongiae	Poecilosclerida	Crellidae	*Crella* sp.	Ascomycota (Fungi)	Henríquez, M. et al. 2014 [5]
15	Demospongiae	Poecilosclerida	Hymedesmiidae	*Kirkpatrickia varialosa*	Crenarchaeota (Archaea); Proteobacteria, Bacteroidetes, Firmicutes	Webster, N.S. et al. 2004 [11]
16	Demospongiae	Poecilosclerida	Hymedesmiidae	*Myxodoryx hanitschi*	Proteobacteria, Bacteroidetes, Actinobacteria, Firmicutes	Savoca, S. et al. 2019; Mangano, S. et al. 2018 [1, 10]
17	Demospongiae	Poecilosclerida	Hymedesmiidae	*Phorbas glaberrimus*	Proteobacteria, Bacteroidetes, Actinobacteria, Firmicutes	Mangano, S. et al. 2018; Savoca, S. et al. 2019 [1, 10]
18	Demospongiae	Poecilosclerida	Latrunculiidae	*Latrunculia apicalis*	Crenarchaeota (Archaea); Proteobacteria, Bacteroidetes, Firmicutes, Planctomyces	Webster, N.S. et al. 2004 [11]
19	Demospongiae	Poecilosclerida	Microcionidae	*Microciona* (*Clathria*)** sp.	Ascomycota (Fungi)	Henríquez, M. et al. 2014; Vaca, I. et al. 2013 [5, 6]
20	Demospongiae	Poecilosclerida	Mycalidae	*Mycale acerata*	Crenarchaeota (Archaea); Proteobacteria, Bacteroidetes, Firmicutes	Webster, N.S. et al. 2004 [11]
21	Demospongiae	Poecilosclerida	Myxillidae	*Myxilla mollis*	Actinobacteria, Firmicutes	Xin Y. et al. 2011 [4]

22	Demospongiae	Polymastiida	Polymastiidae	*Radiella antarctica*	Actinobacteria, Firmicutes	Xin Y. et al. 2011 [4]
23	Demospongiae	Polymastiida	Polymastiidae	*Sphaerotylus antarcticus*	Crenarchaeota (Archaea); Proteobacteria, Bacteroidetes, Firmicutes	Webster, N.S. et al. 2004 [11]
24	Demospongiae	Poecilosclerida	Tedaniidae	*Tedania charcoti*	Proteobacteria, Bacteroidetes, Actinobacteria, Firmicutes	Savoca, S. et al. 2019 [1]
25	Demospongiae	Poecilosclerida	Tedaniidae	*Tedania* sp.	Proteobacteria, Bacteroidetes, Actinobacteria, Firmicutes<br>Ascomycota (Fungi)	Savoca et al. 2019;<br>Henríquez et al. 2014; Vaca et al. 2013 [1, 5, 6]
26	Demospongiae	Poecilosclerida	Tedaniidae	*Trachytedania spinata*	Proteobacteria, Bacteroidetes, Actinobacteria, Firmicutes	Savoca, S. et al. 2019 [1]
27	Demospongiae	Suberitida	Halichondriidae	*Hymeniacidon* sp.	Ascomycota (Fungi)	Henríquez, M. et al. 2014; Vaca, I. et al. 2013 [5, 6]
28	Demospongiae	Suberitida	Suberitidae	*Homaxinella balfourensis*	Crenarchaeota (Archaea); Proteobacteria, Bacteroidetes, Actinobacteria, Firmicutes	Xin Y. et al. 2011; Webster, N.S. et al. 2004 [4, 11]

*The taxonomy has been newly re-identified by Senckenberg researchers as *Anoxycalyx* (*Scolymastra*) *joubini* (D. Janussen).

** Taxonomic changes mean that this is now called *Clathria* (*Microciona*) sp. (WoRMS, 2023)

Table 5. Overview of microbial community diversity of Antarctic sponges revealed by high-throughput sequencing

No.	Class	Order	Family	Genus/ Species	Microbial community diversity (Phylum level *)	References
1	Calcarea	Clathrinida	Leucettidae	*Leucetta antarctica*	20 bacterial phyla; 3 archaeal phyla: Crenarchaeota, Euryarchaeota, Thaumarchaeota; 6 fungal phyla	Moreno-Pino, M. et al. 2020[9]
					11 bacterial phyla; 1 archaeal phylum: Thaumarchaeota; members in Fungi kingdom	Rodríguez-Marconi, S. et al. 2015 [12]
2	Hexactinellida	Lyssacinosida	Rossellidae	*Rossella villosa*	5 bacterial phyla	Papale, M. et al. 2020[13]
3	Hexactinellida	Lyssacinosida	Rossellidae	*Rossella vanhoeffeni*	13 bacterial phyla	Busch et al. 2022[14]
4	Hexactinellida	Lyssacinosida	Rossellidae	*Rossella fibulata*	11 bacterial phyla	Busch et al. 2022[14]
5	Hexactinellida	Lyssacinosida	Rossellidae	*Anoxycalyx joubini*	18 bacterial phyla	Busch et al. 2022[14]
6	Demospongiae	Axinellida	Axinellidae	Axinellidae sp.	22 bacterial phyla	Busch et al. 2022[14]
7	Demospongiae	Dendroceratida	Darwinellidae	*Dendrilla antarctica*	27 bacterial phyla; 3 archaeal phyla: Euryarchaeota, Nanoarchaeota, Thaumarchaeota **	Sacristán-Soriano, O. et al. 2020 [15]
					20 bacterial phyla; 1 archaeal phylum: Thaumarchaeota	Díez-Vives, C. et al. 2020[16]
8	Demospongiae	Haplosclerida	Chalinidae	*Haliclona* (*Rhizoniera*) *dancoi*	6 bacterial phyla	Papale, M. et al. 2020[13]
					9 bacterial phyla	Ruocco, N. et al. 2021[17]
9	Demospongiae	Haplosclerida	Chalinidae	*Haliclona* sp.	14 bacterial phyla	Steinert, G. et al. 2019 [18]
10	Demospongiae	Haplosclerida	Chalinidae	*Haliclona* (*Gellius*) sp.	21 bacterial phyla; 1 archaeal phylum: Thaumarchaeota; members in Fungi kingdom	Rodríguez-Marconi, S. et al. 2015 [12]
11	Demospongiae	Haplosclerida	Chalinidae	*Haliclona (Rhizoniera)* sp.	9 bacterial phyla; 1 archaeal phylum Crenarchaeota	Cristi, A. et al. 2022[19]
12	Demospongiae	Haplosclerida	Niphatidae	*Hemigellius pilosus*	7 bacterial phyla	Papale, M. et al. 2020 [13]
					27 bacterial phyla; 3 archaeal phyla: Euryarchaeota, Nanoarchaeota, Thaumarchaeota **	Sacristán-Soriano, O. et al. 2020 [15]
					6 bacterial phyla; 1 archaeal phylum: Thaumarchaeota	Ruocco, N. et al. 2021[17]
13	Demospongiae	Haplosclerida	Niphatidae	*Microxina sarai*	8 bacterial phyla	Papale, M. et al. 2020[13]
					15 bacterial phyla; 1 archaeal phylum: Thaumarchaeota	Ruocco, N. et al. 2021[17]
14	Demospongiae	Poecilosclerida	Acarnidae	*Megaciella annectens*	16 bacterial phyla; 1 archaeal phylum: Thaumarchaeota; members in Fungi kingdom	Rodríguez-Marconi, S. et al. 2015 [12]

15	Demospongiae	Poecilosclerida	Cladorhizidae	*Cladorhiza* sp.	9 bacterial phyla; 1 archaeal phylum Thaumarchaeota	Georgieva, M.N. et al. 2020[20]
16	Demospongiae	Poecilosclerida	Coelosphaeridae	*Lissodendoryx* (*Ectyodoryx*) *ramilobosa*	5 bacterial phyla	Papale, M. et al. 2020[13]
17	Demospongiae	Poecilosclerida	Hymedesmiidae	*Kirkpatrickia varialosa*	23 bacterial phyla; 2 archaeal phyla: Euryarchaeota, Thaumarchaeota; members in Fungi kingdom	Rodríguez-Marconi, S. et al. 2015 [12]
18	Demospongiae	Poecilosclerida	Hymedesmiidae	*Myxodoryx hanitschi*	4 bacterial phyla: Proteobacteria, Bacteroidetes, Actinobacteria, Firmicutes	Papale, M. et al. 2020 [13]
19	Demospongiae	Poecilosclerida	Hymedesmiidae	*Phorbas* sp.	20 bacterial phyla	Busch, K. et al. 2022[14]
20	Demospongiae	Poecilosclerida	Isodictyidae	*Isodictya bentarti*	13 bacterial phyla	Steinert, G. et al. 2019[18]
21	Demospongiae	Poecilosclerida	Isodictyidae	*Isodictya erinacea*	8 bacterial phyla	Papale, M. et al. 2020[13]
22	Demospongiae	Poecilosclerida	Isodictyidae	*Isodictya kerguelenensis*	22 bacterial phyla; 1 archaea phylum: Thaumarchaeota	Rondon, R. et al. 2020[21]
					9 bacterial phyla; 1 archaeal phylum Crenarchaeota	Cristi, A. et al. 2022[19]
23	Demospongiae	Poecilosclerida	Isodictyidae	*Isodictya* sp.	13 bacterial phyla	Cárdenas, C.A. et al. 2019[22]
24	Demospongiae	Poecilosclerida	Microcionidae	*Clathria* sp.	14 bacterial phyla; 1 archaeal phylum: Thaumarchaeota; members in Fungi kingdom	Rodríguez-Marconi, S. et al. 2015 [12]
25	Demospongiae	Poecilosclerida	Mycalidae	*Mycale* (*Aegogropila*) sp.	4 bacterial phyla: Proteobacteria, Bacteroidetes, Actinobacteria, Firmicutes	Papale, M. et al. 2020[13]
26	Demospongiae	Poecilosclerida	Mycalidae	*Mycale* (*Oxymycale*) *acerata*	12 bacterial phyla	Cárdenas, C.A. et al. 2019[22]
					25 bacterial phyla	Cárdenas, C.A. et al. 2018[23]
					27 bacterial phyla; 3 archaeal phyla: Euryarchaeota, Nanoarchaeota, Thaumarchaeota **	Sacristán-Soriano, O. et al. 2020 [15]
					5 bacterial phyla	Papale, M. et al. 2020[13]
					12 bacterial phyla	Ruocco, N. et al. 2021[17]
					31 bacterial phyla; 3 archaeal phyla: Crenarchaeota; Halobacterota; Nanoarchaeota	Happel, L. et al. 2022[24]
27	Demospongiae	Poecilosclerida	Myxillidae	*Myxilla* (*Burtonanchora*) sp.	16 bacterial phyla; 1 archaeal phylum: Thaumarchaeota; members in Fungi kingdom	Rodríguez-Marconi, S. et al. 2015 [12]
					20 bacterial phyla; 3 archaeal phyla: Crenarchaeota, Euryarchaeota, Thaumarchaeota; 7 fungal phyla	Moreno-Pino, M. et al. 2020[9]

28	Demospongiae	Poecilosclerida	Tedaniidae	*Tedania* (*Tedaniopsis*) *oxeata*	6 bacterial phyla	Papale, M. et al. 2020[13]
29	Demospongiae	Poecilosclerida	Tedaniidae	*Tedania* (*Tedaniopsis*) *wellsae*	12 bacterial phyla	Cárdenas, C.A. et al. 2019[22]
30	Demospongiae	Polymastiida	Polymastiidae	*Sphaerotylus antarcticus*	27 bacterial phyla; 3 archaeal phyla: Euryarchaeota, Nanoarchaeota, Thaumarchaeota **	Sacristán-Soriano, O. et al. 2020 [15]
31	Demospongiae	Polymastiida	Polymastiidae	*Tentorium papillatum*	25 bacterial phyla	Busch, K. et al. 2022[14]
32	Demospongiae	Suberitida	Halichondriidae	*Hymeniacidon torquata*	16 bacterial phyla, 1 archaeal phylum: Thaumarchaeota; members in Fungi kingdom	Rodríguez-Marconi, S. et al. 2015 [12]
					14 bacterial phyla	Cárdenas, C.A. et al. 2019[22]
					10 bacterial phyla; 1 archaeal phylum Crenarchaeota	Cristi, A. et al. 2022
33	Demospongiae	Suberitida	Suberitidae	*Homaxinella balfourensis*	14 bacterial phyla	Steinert, G. et al. 2019 [18]
34	Demospongiae	Tetractinellida	Tetillidae	*Antarctotetilla leptoderma*	21 bacterial phyla	Steinert, G. et al. 2019[18]
35	Demospongiae	Tetractinellida	Tetillidae	*Cinachyra antarctica*	8 bacterial phyla	Papale, M. et al. 2020[13]

*Phyla are listed if the number is less than five.

** The number of the bacterial phyla are revealed from all the sponge species in the study.

Table 6. Prioritisation of Antarctic sponge taxa for microbiome, ecological, and evolutionary research

Host Class/ Order/ Species (or Genus/Family)	Host Importance	Microbiome Importance	Rationale for Prioritisation	Priority	Key References	Availability		
						Sponge	Seq	Micro
**High-level of available data: Identified as having large numbers of records, specimens, and sequences from several publications**								
Demospongiae Dendroceratida ***Dendrilla antarctica***	Widespread in shallow waters, gene flow known, prey–predator dynamics, and unusual seasonal metabolic profile	Diverse with some unique features as well as bioactive compounds	Chemical ecology, gene flow & warming sensitivity	★★	[25-29]			
Demospongiae Tetractinellida ***Antarctotetilla leptoderma***	Widespread, known coloniser, from a taxonomically complex and genetically cryptic group	High levels of diversity with some unique features	Speciation, disturbance & phylogeography	★★	[18, 30, 31]			
Demospongiae Poecilosclerida ***Mycale (Oxymycale) acerata***	Widespread, resilient, fast-growing known coloniser, gene flow known, and with strong metabolic seasonal changes	Spatiotemporally stable microbiome which enable it to be used as a microbiome model	Microbiome structure, gene flow, colonisation & climate resilience	★★★	[11, 26, 32, 33]			
Hexactinellida Lyssacinosida ***Anoxycalyx (Scolymastra) joubini***	Long-lived, structural sponge, which is widespread on the Antarctic shelf but globally limited genus	Moderately diverse microbial associations	Growth, disturbance response, reproduction & habitat structure	★★	[14, 26, 31, 34]			
Hexactinellida Lyssacinosida ***Rossella* spp.**	Habitat-forming hexactinellid with a likely highly endemic species complex	Moderate and distinct microbiomes	Cryptic speciation, benthic structure, disturbance, reproduction, eurybathy & evolution	★★★	[13, 14, 26, 31, 35]			
**Intermediate-level of available data: Identified as having moderate numbers of records, specimens, and sequences from several publications**								
Demospongiae Poecilosclerida **Hymedesmiidae spp. *(Kirkpatrickia, Phorbas)***	Taxonomically diverse and with some host species widespread in either shallower shelf or deeper waters	Moderate to high levels of diversity with some potentially unique features	Cryptic speciation, eurybathy & potential for uncovering evolutionary relationships	★★	[10, 12, 14, 29, 36, 37]			
Demospongiae Poecilosclerida ***Iophon spp.***	Moderately diverse, several species potentially regionally restricted	Exhibits high microbial diversity and unique functional guilds	Microbiome function & ecological role	★★	[9, 28, 29]			
Demospongiae Poecilosclerida ***Isodictya spp.***	Widespread hosts, taxonomically diverse, and with a strong seasonal metabolic profile	Moderate to high levels of diversity with some potentially unique features, and microbiome	Evolutionary divergence, species complexity & environmental stress studies	★★	[21, 26, 29, 33]			

		responds dynamically to temperature changes			
Demospongiae Poecilosclerida **Microcionidae spp.** ***(Artemisina, Clathria)***	Taxonomically diverse and coloniser of ice-scoured substrate	Moderately diverse microbial associations from one species	Unexplored diversity & response to disturbance	★★	[12, 19, 28, 29]
Demospongiae Poecilosclerida ***Myxilla spp.***	Taxonomically diverse, known coloniser, with some host species widespread	Moderate to high levels of microbial diversity found in several species	Microbial biogeography & response to stress and disturbance	★★	[9, 12, 29, 37]
Demospongiae Poecilosclerida ***Tedania spp.***	Relatively widespread species, moderately diverse, but likely understudied taxon	Specialized microbial communities found despite low to moderate levels of diversity	Unexplored diversity & functional potential of rare taxa	★★	[1, 13, 28, 35]
Demospongiae Polymastiida ***Sphaerotylus antarcticus***	Widespread, potential microhabitat provider, with a strong seasonal metabolic profile	Understudied but has a microbiome which needs more research	Habitat-forming & potential indicator of benthic change	★★	[11, 15]
Demospongiae Polymastiida ***Tentorium papillatum***	Widespread but with limited global diversity	Diverse with some unique features	Insight into sponge evolution & endemism	★★	[14, 35]
Demospongiae Suberitida ***Homaxinella balfourensis***	Widespread coloniser of ice-scoured environments with a strong seasonal metabolic profile	Understudied microbiome that could indicate novelty in this pioneer	Monitoring environmental stress & disturbance response	★★	[11, 18, 38, 39]
Demospongiae Tetractinellida ***Cinachyra spp.***	Widespread, known coloniser, from a taxonomically complex and genetically cryptic group	Moderate diversity with some unique features	Microbiome function of a slow-growing organism, evolution & disturbance	★★	[13, 26, 30, 31, 35]
**Low-level of available data: Identified as having either limited records, specimens and/or sequences from several publications**					
Calcarea/ Clathrinida **Clathrinida spp. (Clathrinidae, Dendyidae, Leucascididae, Leucettidae)**	Taxonomically diverse, several endemic genera, and understudied apart from *Leucetta antarctica*	Unique and diverse features found which could be used to track environmental change	Ocean acidification, reproduction & microbiome response important to monitor	★★★	[11, 12, 26, 40, 41]
Calcarea Leucosolenida **Leucosolenida spp.** ***(Achramorpha, Grantia, Jenkina, Leucandra, Megapogon, Sycon)***	Taxonomically diverse, several endemic genera, and understudied	Unique and diverse features found which could be used to track environmental change	Ocean acidification, reproduction & microbiome response important to monitor	★★★	[11, 26, 40, 41]

Demospongiae Axinellida **Axinellidae spp.** ***(Axinella, Phakellia)***	Taxonomically diverse under-studied family	Moderate levels of diversity but research is limited	Potential indicator of environmental gradients on deep shelf environments & phylogenetic divergence and glacial refugia	★	[42]	amber	amber	amber
Demospongiae Haplosclerida ***Haliclona spp.***	Taxonomic diversity with some host species widespread at shallower depths	Moderate level diversity and some unique elements, with a potential for symbiotic specialization	Symbiosis evolution & unique microbial associations	★★	[12, 13, 18, 26, 29, 31, 37]	green	amber	amber
Demospongiae Poecilosclerida **Cladorhizidae spp.**	Carnivorous, taxonomically diverse, and occupies a deep-sea niche	Unusual bacteria have been found which support carnivorous sponges	Trophic ecology & deep-sea specialisation	★★	[20, 26, 43]	green	green	red
Demospongiae Suberitida ***Stylocordyla chupachups***	Forms dense populations correlated with areas of high productivity and has a strong seasonal metabolic profile	No known microbiome studies, but could be important in Suberitida evolution	Trophic innovation, reproduction & colonisation	★★	[44]	green	amber	red
Hexactinellida Amphidiscosida ***Hyalonema*** **spp.**	Taxonomically diverse, several endemic species, and locally abundant in some deep-sea environments	Microbiome data sparse globally; potential for symbiotic archaea or chemosymbionts	Deep benthic adaptation & evolution	★	[45]	amber	red	red
Hexactinellida Lyssacinosida **Lyssacinosida spp. (Euplectellidae and other Rossellidae -** ***Bathydorus, Caulphacus)***	Structurally important in deep-sea habitats, taxonomcally diverse with many endemic species	Microbiome data sparse globally; potential for symbiotic archaea or chemosymbionts	Deep benthic adaptation & evolution	★★	[26]	green	amber	red
Hexactinellida Sceptrulophora **Sceptrulophora spp.** ***(Chonelasma, Lonchiphora)***	Less well-known group of deep-sea hexactinellids, with an increasing understanding of taxonomic diversity	Microbiome data sparse globally; potential for symbiotic archaea or chemosymbionts	Deep benthic adaptation & evolution	★	[26, 46]	amber	amber	red
Homoscleromorpha Homosclerophorida **Plakinidae spp.**	Rare around Antarctica with few species found	Known for diverse symbiotic microbes elsewhere but Antarctic forms understudied	Polar adaptation & basal lineage in sponge phylogeny	★★	Costa et al. (2022)[47]	amber	green	red

Note: These hosts were selected based on their ecological importance, evolutionary interest, or unique and understudied microbiomes. A three-star priority system indicates which groups should be studied first. Data availability for each taxon is shown using colour codes: green (high), amber (moderate), and red (low), based on existing records from OBIS and genetic databases (BOLD, GenBank, EMBL-EBI, SBD).

Reference

1. Savoca S, Lo Giudice A, Papale M, Mangano S, Caruso C, Spanò N, Michaud L, Rizzo C (2019) Antarctic sponges from the Terra Nova Bay (Ross Sea) host a diversified bacterial community. Scientific Reports 9: 16135. doi: 10.1038/s41598-019-52491-0
2. Papaleo MC, Fondi M, Maida I, Perrin E, Lo Giudice A, Michaud L, Mangano S, Bartolucci G, Romoli R, Fani R (2012) Sponge-associated microbial Antarctic communities exhibiting antimicrobial activity against Burkholderia cepacia complex bacteria. Biotechnology Advances 30: 272-293. doi: https://doi.org/10.1016/j.biotechadv.2011.06.011
3. Mangano S, Michaud L, Caruso C, Brilli M, Bruni V, Fani R, Lo Giudice A (2009) Antagonistic interactions between psychrotrophic cultivable bacteria isolated from Antarctic sponges: a preliminary analysis. Research in Microbiology 160: 27-37. doi: https://doi.org/10.1016/j.resmic.2008.09.013
4. Xin Y, Kanagasabhapathy M, Janussen D, Xue S, Zhang W (2011) Phylogenetic diversity of Gram-positive bacteria cultured from Antarctic deep-sea sponges. Polar Biology 34: 1501-1512. doi: 10.1007/s00300-011-1009-y
5. Henríquez M, Vergara K, Norambuena J, Beiza A, Maza F, Ubilla P, Araya I, Chávez R, San-Martín A, Darias J, Darias MJ, Vaca I (2014) Diversity of cultivable fungi associated with Antarctic marine sponges and screening for their antimicrobial, antitumoral and antioxidant potential. World Journal of Microbiology and Biotechnology 30: 65-76. doi: 10.1007/s11274-013-1418-x
6. Vaca I, Faúndez C, Maza F, Paillavil B, Hernández V, Acosta F, Levicán G, Martínez C, Chávez R (2013) Cultivable psychrotolerant yeasts associated with Antarctic marine sponges. World Journal of Microbiology and Biotechnology 29: 183-189. doi: 10.1007/s11274-012-1159-2
7. Orlandini V, Maida I, Fondi M, Perrin E, Papaleo MC, Bosi E, de Pascale D, Tutino ML, Michaud L, Lo Giudice A, Fani R (2014) Genomic analysis of three sponge-associated Arthrobacter Antarctic strains, inhibiting the growth of Burkholderia cepacia complex bacteria by synthesizing volatile organic compounds. Microbiological Research 169: 593-601. doi: https://doi.org/10.1016/j.micres.2013.09.018
8. Mangano S, Michaud L, Caruso C, Lo Giudice A (2014) Metal and antibiotic resistance in psychrotrophic bacteria associated with the Antarctic sponge Hemigellius pilosus (Kirkpatrick, 1907). Polar Biology 37: 227-235. doi: 10.1007/s00300-013-1426-1
9. Moreno-Pino M, Cristi A, Gillooly JF, Trefault N (2020) Characterizing the microbiomes of Antarctic sponges: a functional metagenomic approach. Scientific reports 10: 1-12.
10. Mangano S, Caruso C, Michaud L, Lo Giudice A (2018) First evidence of quorum sensing activity in bacteria associated with Antarctic sponges. Polar Biology 41: 1435-1445. doi: 10.1007/s00300-018-2296-3
11. Webster N, Negri A, Munro M, Battershill C (2004) Diverse microbial communities inhabit Antarctic sponges. Environ Microbiol 6: 288-300.
12. Rodríguez-Marconi S, De la Iglesia R, Díez B, Fonseca CA, Hajdu E, Trefault N (2015) Characterization of bacterial, archaeal and eukaryote symbionts from Antarctic sponges reveals a high diversity at a three-domain level and a particular signature for this ecosystem. PLoS One 10: e0138837.

13. Papale M, Rizzo C, Fani R, Bertolino M, Costa G, Paytuví-Gallart A, Schiaparelli S, Michaud L, Azzaro M, Lo Giudice A (2020) Exploring the diversity and metabolic profiles of bacterial communities associated with Antarctic sponges (Terra Nova Bay, Ross Sea). Frontiers in Ecology and Evolution: 268.
14. Busch K, Slaby BM, Bach W, Boetius A, Clefsen I, Colaço A, Creemers M, Cristobo J, Federwisch L, Franke A, Gavriilidou A, Hethke A, Kenchington E, Mienis F, Mills S, Riesgo A, Ríos P, Roberts EM, Sipkema D, Pita L, Schupp PJ, Xavier J, Rapp HT, Hentschel U (2022) Biodiversity, environmental drivers, and sustainability of the global deep-sea sponge microbiome. Nat Commun 13: 5160. doi: 10.1038/s41467-022-32684-4
15. Sacristán-Soriano O, Pérez Criado N, Avila C (2020) Host species determines symbiotic community composition in Antarctic sponges (Porifera: Demospongiae). Frontiers in Marine Science 7: 474.
16. Díez-Vives C, Taboada S, Leiva C, Busch K, Hentschel U, Riesgo A (2020) On the way to specificity-Microbiome reflects sponge genetic cluster primarily in highly structured populations. Molecular ecology 29: 4412-4427.
17. Ruocco N, Esposito R, Bertolino M, Zazo G, Sonnessa M, Andreani F, Coppola D, Giordano D, Nuzzo G, Lauritano C (2021) A metataxonomic approach reveals diversified bacterial communities in antarctic sponges. Marine drugs 19: 173.
18. Steinert G, Wemheuer B, Janussen D, Erpenbeck D, Daniel R, Simon M, Brinkhoff T, Schupp PJ (2019) Prokaryotic diversity and community patterns in Antarctic continental shelf sponges. Frontiers in Marine Science 6: 297.
19. Cristi A, Parada-Pozo G, Morales-Vicencio F, Cárdenas CA, Trefault N (2022) Variability in Host Specificity and Functional Potential of Antarctic Sponge-Associated Bacterial Communities. Frontiers in Microbiology: 4024.
20. Georgieva MN, Taboada S, Riesgo A, Díez-Vives C, De Leo FC, Jeffreys RM, Copley JT, Little CT, Ríos P, Cristobo J (2020) Evidence of vent-adaptation in sponges living at the periphery of hydrothermal vent environments: ecological and evolutionary implications. Frontiers in microbiology: 1636.
21. Rondon R, González-Aravena M, Font A, Osorio M, Cárdenas CA (2020) Effects of Climate Change Stressors on the Prokaryotic Communities of the Antarctic Sponge Isodictya kerguelenensis. Frontiers in Ecology and Evolution: 262.
22. Cárdenas CA, Font A, Steinert G, Rondon R, González-Aravena M (2019) Temporal stability of bacterial communities in Antarctic sponges. Frontiers in microbiology: 2699.
23. Cárdenas CA, González-Aravena M, Font A, Hestetun JT, Hajdu E, Trefault N, Malmberg M, Bongcam-Rudloff E (2018) High similarity in the microbiota of cold-water sponges of the Genus Mycale from two different geographical areas. PeerJ 6: e4935.
24. Happel L, Rondon R, Font A, González-Aravena M, Cárdenas CA (2022) Stability of the Microbiome of the Sponge Mycale (Oxymycale) acerata in the Western Antarctic Peninsula. Front Microbiol 13: 827863. doi: 10.3389/fmicb.2022.827863
25. McClintock JB, Amsler CD, Baker BJ, van Soest RWM (2005) Ecology of Antarctic marine sponges: an overview. Integrative and Comparative Biology 45: 359-368.

26. Janussen D, Downey RV (2014) Biogeographic atlas of the Southern Ocean. In: De Broyer, C, Koubbi, P, Griffiths, HJ, Raymond, B, Udekem d'Acoz, Cd (eds.) Porifera. Cambridge: Scientific Committee on Antarctic Research., pp. 94-102
27. Leiva C, Taboada S, Kenny NJ, Combosch D, Giribet G, Jombart T, Riesgo A (2019) Population substructure and signals of divergent adaptive selection despite admixture in the sponge Dendrilla antarctica from shallow waters surrounding the Antarctic Peninsula. Molecular Ecology 28: 3151-3170.
28. Freitas MAMd, Cunha-Ferreira IC, Leal CV, Fernandez JCC, Omachi CY, Campos LS, Masi BP, Krüger RH, Hajdu E, Thompson CC, Thompson FL (2023) Microbiome diversity from sponges biogeographically distributed between South America and Antarctica. Science of The Total Environment 879: 163256. doi: https://doi.org/10.1016/j.scitotenv.2023.163256
29. Moreno-Pino M, Manrique-de-la-Cuba MF, López-Rodríguez M, Parada-Pozo G, Rodríguez-Marconi S, Ribeiro CG, Flores-Herrera P, Guajardo M, Trefault N (2024) Unveiling microbial guilds and symbiotic relationships in Antarctic sponge microbiomes. Scientific Reports 14: 6371. doi: 10.1038/s41598-024-56480-w
30. Carella M, Agell G, Cárdenas P, Uriz MJ (2016) Phylogenetic reassessment of Antarctic Tetillidae (Demospongiae, Tetractinellida) Reveals New Genera and Genetic Similarity among Morphologically Distinct Species. PLoS ONE 11: e0160718.
31. Dayton PK, Jarrell SC, Kim S, Ed Parnell P, Thrush SF, Hammerstrom K, Leichter JJ (2019) Benthic responses to an Antarctic regime shift: food particle size and recruitment biology. Ecological Applications 29: e01823.
32. Barnes DK, Sands CJ, Hogg OT, Robinson BJ, Downey RV, Smith JA (2016) Biodiversity signature of the last glacial maximum at South Georgia, Southern Ocean. Journal of Biogeography 43: 2391-2399.
33. Cárdenas CA, González-Aravena M, Font A, Hestetun JT, Hajdu E, Trefault N, Malmberg M, Bongcam-Rudloff E (2018) High similarity in the microbiota of cold-water sponges of the Genus Mycale from two different geographical areas. PeerJ 6.
34. Dayton PK, Kim S, Jarrell SC, Oliver JS, Hammerstrom K, Fisher JL, O'Connor K, Barber JS, Robilliard G, Barry J (2013) Recruitment, growth and mortality of an Antarctic hexactinellid sponge, Anoxycalyx joubini. PloS one 8: e56939.
35. Vargas S, Kelly M, Schnabel K, Mills S, Bowden D, Wörheide G (2015) Diversity in a Cold Hot-Spot: DNA-Barcoding Reveals Patterns of Evolution among Antarctic Demosponges (Class Demospongiae, Phylum Porifera). PLoS ONE 10: e0127573.
36. Koutsouveli V, Taboada S, Moles J, Cristobo J, Ríos P, Bertran A, Solà J, Avila C, Riesgo A (2018) Insights into the reproduction of some Antarctic dendroceratid, poecilosclerid, and haplosclerid demosponges. PLoS One 13: e0192267.
37. Manrique-de-la-Cuba MF, Parada-Pozo G, Rodríguez-Marconi S, López-Rodríguez MR, Abades S, Trefault N (2024) Evidence of habitat specificity in sponge microbiomes from Antarctica. Environmental Microbiome 19: 100. doi: 10.1186/s40793-024-00648-4
38. Dayton PK (1989) Interdecadal variation in an Antarctic sponge and its predators from oceanographic climate shifts. Science 245: 1484-1486.

39. McClintock JB, Baker BJ, Slattery M, Hamann M, Kopitzke R, Heine J (1994) Chemotactic tube-foot responses of a spongivorous sea star Perknaster fuscus to organic extracts from antarctic sponges. Journal of Chemical Ecology 20: 859-870.
40. McClintock JB, Amsler CD, Baker BJ, Van Soest RW (2005) Ecology of Antarctic marine sponges: an overview. Integr Comp Biol 45: 359-368.
41. Riesgo A, Cavalcanti FF, Kenny NJ, Ríos P, Cristobo J, Lanna E (2018) Integrative systematics of clathrinid sponges: morphological, reproductive and phylogenetic characterisation of a new species of Leucetta from Antarctica (Porifera, Calcarea, Calcinea) with notes on the occurrence of flagellated sperm. Invertebrate Systematics 32: 827-841.
42. Díez-Vives C, Koutsouveli V, Conejero M, Riesgo A (2022) Global patterns in symbiont selection and transmission strategies in sponges. Frontiers in Ecology and Evolution Volume 10 - 2022. doi: 10.3389/fevo.2022.1015592
43. Vacelet J, Boury-Esnault N, Fiala-Medioni A, Fisher CR (1995) A methanotrophic carnivorous sponge. Nature 377: 296.
44. Carella M, Agell G, Uriz MJ (2019) Asexual reproduction and heterozygote selection in an Antarctic demosponge (Stylocordyla chupachus, Suberitida). Polar Biology 42: 475-483.
45. Beaulieu SE (2001) Life on glass houses: sponge stalk communities in the deep sea. Marine Biology 138: 803-817. doi: 10.1007/s002270000500
46. Janussen D, Tendal OS (2007) Diversity and distribution of Porifera in the bathyal and abyssal Weddell Sea and adjacent areas. Deep-Sea Research II 54: 1864-1875.
47. Costa G, Bavestrello G, Canese S, Canessa M, Mazzoli C, Montagna P, Puce S, Schiaparelli S, Bertolino M (2022) Sponges associated with stylasterid thanatocoenosis (Cnidaria, Hydrozoa) from the deep Ross Sea (Southern Ocean). Polar Biology 45: 703-718. doi: 10.1007/s00300-022-03023-6
